# Hepatopulmonary syndrome in patients with porto-sinusoidal vascular disorder: Characteristics and outcome^☆^

**DOI:** 10.1016/j.jhepr.2024.101310

**Published:** 2024-12-20

**Authors:** Sabrina Sidali, Ylang Spaes, Kinan El Husseini, Odile Goria, Vincent Mallet, Armelle Poujol-Robert, Anne Gervais, Adrien Lannes, Dominique Thabut, Jean-Baptiste Nousbaum, Isabelle Hourmand-Ollivier, Charlotte Costentin, Alexandra Heurgué, Pauline Houssel-Debry, Sophie Hillaire, Nathalie Ganne-Carrié, Nicolas Drilhon, Shanta Ram Valainathan, Lucile Moga, Marion Tanguy, Estelle Marcault, Aurélie Plessier, François Durand, Sarah Raevens, Valérie Paradis, Agnès Cachier, Laure Elkrief, Pierre-Emmanuel Rautou

**Affiliations:** 1 Centre de Recherche sur l'Inflammation, Université Paris-Cité, Inserm, Paris, France; 2 AP-HP, Hôpital Beaujon, Service d'Hépatologie, DMU DIGEST, Centre de Référence des Maladies Vasculaires du Foie, FILFOIE, ERN RARE-LIVER, Clichy, France; 3 Hépato-Gastroentérologie, Centre Hospitalier Universitaire Charles Nicolle, Rouen, France; 4 APHP, Service de Pneumologie, Centre de Référence des Maladies Pulmonaires Rares, FHU APOLLO, Hôpital Bichat, Paris, France; 5 Hôpital Cochin, AP-HP, Hépatologie, Paris, France; 6 Hôpital Saint-Antoine, AP-HP, Hépatologie, Paris, France; 7 Hôpital Louis-Mourier, AP-HP, Hépato-gastroentérologie, Paris, France; 8 Centre Hospitalier Universitaire Angers, Hépatologie, Angers, France; 9 Service d'Hépato-gastroentérologie, Hôpital Universitaire Pitié-Salpêtrière, AP-HP Sorbonne Université, Paris, France; 10 Institute of Cardiometabolism and Nutrition, INSERM, Centre de Recherche Saint-Antoine, Sorbonne Université, Paris, France; 11 Centre Hospitalier Régional Universitaire Morvan, Hépatologie, Brest, France; 12 Centre Hospitalier Universitaire de Caen Normandie, Hépatologie, Caen, France; 13 Hepato-Gastroenterology and Digestive Oncology Department, CHU Grenoble Alpes / Institute for Advanced Biosciences, CNRS UMR 5309-INSERM U1209, University Grenoble Alpes, Grenoble, France; 14 Hépatologie, Centre Hospitalier Universitaire de Reims, Reims, France; 15 Hépatologie, Centre Hospitalier Universitaire, Rennes, France; 16 Hépatologie, Hôpital Foch, Suresnes, France; 17 Liver Unit, Hôpital Avicenne, Hôpitaux Universitaires Paris-Seine-Saint-Denis, Assistance Publique Hôpitaux de Paris, Bobigny, France; 18 Unité de Formation et de Recherche Santé Médecine et Biologie Humaine, Université Sorbonne Paris Nord, Bobigny, France; 19 AP-HP, Hôpital Bichat, Unité de Recherche Clinique Nord Secteur Ouest, Paris, France; 20 Department of Gastroenterology and Hepatology, Ghent University, Ghent University Hospital, Ghent, Belgium; 21 Département de Pathologie, Hôpital Beaujon, AP-HP Nord, UPC, Clichy, France; 22 Université Paris-Cité, Department of Cardiology, Bichat/Beaujon Hospital (AP-HP Nord), ENETS Centre of Excellence, Paris, Clichy, France; 23 Hépato-gastroéntérologie, Hôpital Trousseau, Centre Hospitalier Régional Universitaire, Tours, France; 24 Faculté de Médecine de Tours, University of Tours, Tours, France

**Keywords:** Hepatopulmonary syndrome, Hypoxemia, Liver, Lung–liver interaction, Portal hypertension, Vascular liver disease

## Abstract

**Background & Aims:**

Porto-sinusoidal vascular disorder (PSVD) is a rare cause of portal hypertension. Data on hepatopulmonary syndrome (HPS) in PSVD are limited. This study aimed to determine the associated factors, plasma mediators, and evolution of HPS in patients with PSVD.

**Methods:**

Multicenter observational study of patients with PSVD with signs of portal hypertension in whom contrast-enhanced transthoracic echocardiography (CE-TTE) was performed.

**Results:**

Among 196 patients with PSVD who underwent CE-TTE in 17 centers, 14 (7% [95% confidence interval 3–11%]) had a confirmed diagnosis of HPS. Patients with HPS more frequently had a genetic disorder associated with PSVD (50% *vs.* 6%, *p* <0.001), especially telomere biology disorders (*p* <0.001). Liver function was less preserved in patients with HPS, because they had lower prothrombin index (63% *vs.* 86%, *p* = 0.04), higher serum total bilirubin (37 μmol/L *vs.* 14 μmol/L, *p* <0.001), and lower serum albumin (32 g/L *vs.* 38 g/L, *p* <0.001). HPS tended to be associated with more portal venule obliterations (*p* = 0.085) and with nodular liver architecture (*p* = 0.069). Plasma concentrations of Angiopoietin-2, ICAM3, and Tie2 were higher in patients with HPS (*p* = 0.02, *p* = 0.04, *p* = 0.01, respectively). Out of the 14 patients with HPS, five underwent liver transplantation after a median follow-up of 34 months. Overall cumulative incidence of liver-related events and of death was similar between patients with and without HPS, when considering liver transplantation for HPS as a competing risk.

**Conclusions:**

HPS in patients with PSVD was associated with genetic disorders, less preserved liver function, and higher plasma concentrations of angiogenic mediators. When applying HPS model for end-stage liver disease exception policy for liver transplantation, overall survival of patients with PSVD and HPS was similar to that of patients with PSVD without HPS.

**Impact and implications:**

Hepatopulmonary syndrome (HPS) is a rare complication of porto-sinusoidal vascular disorder (PSVD). This multicentric study found that patients with PSVD and HPS had less preserved liver function, and harbored genetic disorders more frequently (especially telomere biology disorders) than patients without HPS. HPS did not negatively impact transplantation-free survival when applying HPS MELD exception policy for liver transplantation through a competitive risk analysis. Our findings suggest that patients with PSVD with respiratory symptoms and/or telomere biology disorders may benefit from systematic screening for HPS.

## Highlights

• Out of 196 patients with PSVD screened for HPS, 14 (7%) had HPS.
• HPS was associated with genetic disorders, particularly telomere biology disorders, and features suggesting worse liver function.
• Plasma concentrations of angiogenic mediators were elevated in patients with HPS.
• When applying MELD exception policy for LT, HPS was not associated with survival in patients with PSVD.
• Screening for HPS should be considered in patients with PSVD and respiratory symptoms.

## Graphical abstract

**Figure:**
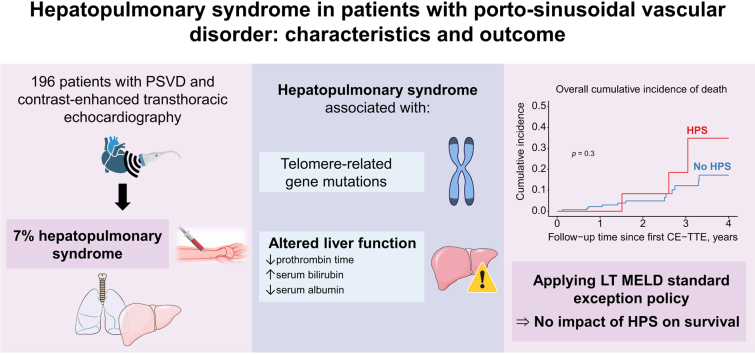

## Introduction

Porto-sinusoidal vascular disorder (PSVD) encompasses a heterogeneous group of rare liver diseases characterized by abnormalities of the small intrahepatic vessels in the absence of cirrhosis.^1^^,^^2^ PSVD has been associated with various conditions, including thrombophilia, hematologic malignancies, HIV infection, genetic disorders, and immunological disorders.^1^^,^[3], [4], [5], [6] The main complications of PSVD are portal venous thrombosis[5], [6], [7], [8] and gastrointestinal bleeding related to portal hypertension.^3^^,^^5^^,^^6^ Development of hepatic encephalopathy^5^^,^^6^ and refractory ascites^5^^,^^6^ are uncommon, and hepatocellular carcinoma exceptional.^1^

Hepatopulmonary syndrome (HPS) is characterized by intrapulmonary vascular dilatations and an increased alveolar–arterial gradient (A–a gradient) in patients with chronic liver disease and/or portal hypertension.^9^ If the pathogenesis of HPS is not fully understood, pulmonary endothelial dysfunction and angiogenesis, bacterial translocation with pulmonary intravascular recruitment of immune cells, and alveolar type II (AT2) cell dysfunction represent the most important identified mechanisms.^10^ In this regard, increased plasma concentrations of several angiogenic mediators, including angiopoietin 2, TEK tyrosine kinase endothelial (Tie2), intercellular adhesion molecule 3 (ICAM3), and vascular cell adhesion molecule 1 (VCAM1), are increased in patients with HPS and cirrhosis.^11^^,^^12^ Moreover, inflammatory mediators, including tissue necrosis factor alpha (TNF-α), may also be associated with the development of HPS.^10^^,^^13^ Regarding management, oxygen supplementation is used as a symptomatic treatment, particularly in cases of severe hypoxemia at rest or oxygen desaturation during exercise. There is currently no drug therapy available for the management of HPS, and the only effective treatment appears to be liver transplantation (LT).^9^ Given the unfavorable prognosis without LT, the diagnosis of HPS associated with a partial pressure of oxygen <60 mmHg is considered as a priority indication for LT, with model for end-stage liver disease (MELD) exception policy, assuming no other abnormality contributing to hypoxemia.^9^^,^^14^^,^^15^

HPS has been reported in patients with PSVD (Table S1). However, available data are mostly derived from isolated case reports. Only two series gathering 19 and 24 patients with PSVD each described two patients with HPS.^16^^,^^17^ Therefore, we currently lack reliable information on HPS in patients with PSVD.

The aim of this study was to improve our understanding of HPS associated with PSVD by determining (a) clinical, laboratory and histological features associated with HPS in PSVD; (b) plasma angiogenic and inflammatory mediators associated with HPS in PSVD; (c) outcomes according to HPS in PSVD.

## Patients and methods

### Study cohort

This multicenter observational study was a retrospective analysis of prospectively collected data on all patients with PSVD and signs of portal hypertension, who underwent liver biopsy between 2000 and 2022 and contrast-enhanced transthoracic echocardiography (CE-TTE) between 2014 and 2023, in one of the centers of the French network for vascular liver diseases. Indeed, since 2014, screening for HPS and CE-TTE has been part of the routine investigations carried out when patients are referred for PSVD to the French reference center for vascular liver diseases (Hôpital Beaujon, Clichy, France) and when patients are included into the multicenter APIS randomized controlled trial (NCT04007289).

Diagnosis of PSVD was based on Vascular Liver Disease Interest Group (VALDIG) criteria, as stated in the Baveno VII consensus:^2^ adequate liver biopsy (≥20 mm long and not too fragmented, or considered adequate for interpretation by an expert pathologist) or liver explant without cirrhosis, and one sign specific for portal hypertension; or adequate liver biopsy or liver explant without cirrhosis and one sign not specific for portal hypertension and one histological lesion not specific for PSVD. Specific signs of portal hypertension included gastric, esophageal, or ectopic varices, history of portal hypertensive bleeding, and portosystemic collaterals at imaging. Non-specific signs of portal hypertension included ascites, platelet count <150,000/mm^3^, and spleen size ≥13 cm in the largest axis.^2^ The date of PSVD diagnosis was the date of the liver biopsy basing PSVD diagnosis.

Non-inclusion criteria were other causes of portal hypertension (Budd–Chiari syndrome, cardiac insufficiency, Fontan surgery, hereditary hemorrhagic telangiectasia, Abernethy syndrome, chronic cholestatic disease, liver infiltration by tumor cells, hepatic schistosomiasis diagnosed on liver biopsy), cavernoma or complete portal vein thrombosis at the time of liver biopsy, lack of data, and absence of patient consent.

The protocol was performed in accordance with ethical guidelines of the 1975 Declaration of Helsinki and was approved by the institutional review board (CPP Ile de France IV, IRB n 2003/21, Paris, France). None of the patients included in this study refused permission for use of their case records for medical research. This observational cohort study was designed, conducted, and written following the STROBE guidelines.

### Diagnosis of hepatopulmonary syndrome

HPS diagnosis was based on the following criteria in patients with PSVD and signs of portal hypertension: (i) abnormal arterial oxygenation attested by an elevated alveolar–arterial oxygen gradient (AaPO_2_) (≥15 mmHg in room air in patients aged <65 years, and ≥20 mmHg in patients aged ≥65 years) and/or an oxygen partial pressure (PaO_2_) <80 mmHg, and (ii) CE-TTE showing the appearance of microbubbles in the left heart chambers three to six cycles after right atrial passage, reflecting intrapulmonary vascular dilatations.^18^^,^^19^

### Collected data

Clinical, laboratory, endoscopic, and imaging data were collected at the time of liver biopsy, at the time of CE-TTE, and at the end of follow-up.

Collected patients’ characteristics included risk factors for chronic liver disease, namely history of arterial hypertension, diabetes, dyslipidemia, overweight defined as BMI ≥25 kg/m^2^, hepatitis B and C virus infection markers, excessive alcohol consumption defined as more than 14 glasses per week for women and more than 21 glasses per week for men. Extrahepatic conditions associated with PSVD were searched for, namely immunological disorders (immune deficiency, autoimmune conditions, history of solid organ transplantation, inflammatory bowel disease), HIV infection, medication or toxins (in particular azathioprine, chemotherapy, didanosine), hematological disease and prothrombotic conditions (myeloproliferative or lymphoproliferative syndromes, thrombophilia, antiphospholipid syndrome), or genetic disorder (especially telomere biology disorder). Other comorbidities, including cardiovascular and pulmonary comorbidities, were also noted.

Current ascites or a history of ascites were defined by either clinically detectable ascites or ascites controlled using diuretic therapy. Onset or worsening of ascites was defined as onset of clinically detectable ascites confirmed by imaging in patients without previous ascites, and as ascites requiring ≥2 paracentesis procedures or requiring a transjugular intrahepatic portosystemic shunt (TIPS) in patients with previous ascites not requiring paracentesis. Hepatic encephalopathy was assessed according to the West Haven criteria.

Portosystemic collaterals and spleen size were assessed using liver ultrasonography, computed tomography (CT) scan, or magnetic resonance imaging (MRI) studies performed within 1 year of liver biopsy (PSVD diagnosis), and within 1 year of CE-TTE.

Liver and spleen stiffness were measured by vibration-controlled transient elastography (FibroScan^TM^, Echosens, Paris, France), within 6 months before or after CE-TTE.

Endoscopic data were obtained from upper gastrointestinal endoscopies performed within 1 year before or after liver biopsy, for PSVD diagnosis, and within 1 year before or after the CE-TTE, for patients’ description, or >1 year before CE-TTE in patients not requiring endoscopic control. The presence and size of gastro-esophageal varices and history of variceal band ligation or glue were collected. Varices at risk were defined as large varices and/or history of band ligation or glue.

Liver-related events were defined as onset or worsening of ascites, spontaneous bacterial peritonitis, onset or worsening of hepatic encephalopathy, gastrointestinal bleeding caused by portal hypertension, or portal venous thrombosis development or progression.

In France, since 2010, HPS associated with an oxygen partial pressure of <60 mmHg is considered as a priority indication for LT, with MELD exception policy, assuming no other abnormality contributing to hypoxemia.^15^ Patients fulfilling those criteria are given access to transplantation within 3 months; no priority for LT is given for patients with a PaO_2_ ≥60 mmHg and re-evaluation is proposed.

### Histological analysis

Liver biopsies were reviewed by an expert pathologist (VP) with expertise in vascular liver diseases – unaware of clinical, laboratory, imaging, and endoscopic data – according to predetermined criteria and classification previously reported.^20^

### Measurement of plasma concentrations of angiogenic and inflammatory mediators and of endotoxin

Based on available knowledge on the pathophysiology of HPS in cirrhosis,[10], [11], [12], [13], [9] we measured concentrations of angiogenic and inflammatory mediators in plasma samples from patients with PSVD without and with HPS, as well as in plasma samples from patients with cirrhosis without and with HPS and from healthy individuals, as reference. In all groups, peripheral venous blood was collected from the cubital vein, with a tourniquet needle, in 0.129 mol/L citrated tubes. Two successive centrifugations were performed, each of 15 min at 2,500 × *g* at 20 °C. Aliquots of platelet-free plasma were then stored at -80 °C until use.

For patients with PSVD, blood was collected at the time of CE-TTE or within 12 months before or after CE-TTE. Patients with cirrhosis and HPS were identified within the previously published MICROSPY cohort^21^ and compared with twice as many patients with cirrhosis without HPS, randomly selected from the same cohort.

We measured concentrations of angiopoietin 2 (DY623 DY008; R&D Systems, Minneapolis, USA), Tie2 (DY5159, DY008; R&D Systems, Minneapolis, USA), ICAM3 (DY715, DY008, R&D Systems, Minneapolis, USA), VCAM1 (DY809-05, DY008; R&D Systems, Minneapolis, USA), IL-6 (DY206-05, DY008; R&D Systems, Minneapolis, USA), and TNF-α (DY210-05, DY008; R&D Systems, Minneapolis, USA) according to the manufacturer’s instructions. The chromogenic limulus amoebocyte lysate assay (Endochrome-K test R1708K; Charles River Laboratories, Charleston, SC, USA) was used for the detection of endotoxin. For optimal test results, platelet-free plasmas were diluted 1:10 with endotoxin-free water and heat-treated for 30 min at 75 °C. Samples were then mixed with limulus amoebocyte lysate reagent and absorbance of the plate (405 nm) then read for 1 h using a kinetic microplate reader (Tecan Spark 10M, Tecan Austria GmbH, Grödig, Austria) and analyzed. To reduce interassay variability, all samples were measured as a single batch.

### Patients and public involvement

Patients or the public were not involved in the design, conduct, or reporting of our research. The French Association of Vascular Liver Disease Patients (AMVF) has contributed financially to the APIS study and will disseminate the results of this study when they are published.

### Statistical analysis

Quantitative variables were expressed as median (interquartile ranges) and were compared using the Mann–Whitney *U* test, unless otherwise stated. Qualitative variables were expressed as absolute and relative (percentage) frequencies and compared using the Χ^2^ test or Fisher’s exact test when appropriate.

A backward stepwise logistic regression was used to identify independent predictors of HPS. At each step, variables were chosen based on *p* values and the Akaike Information Criterion was used to set a limit on the total number of variables included in the final model.

Overall cumulative incidence of death from the date of CE-TTE was assessed using the Kaplan–Meier method and comparison between patients with and without HPS was performed using the log-rank test. HPS being an indication for LT *per se*, we also assessed patients’ outcome from the date of CE-TTE using a multistate model: patients alive without LT were censored at the date of the last follow-up visit and coded 0; patients who died before LT or who underwent LT not indicated for HPS were counted as event at the date of LT or death, whichever occurred first, and coded 1; LT for HPS was considered to be a competing event, and coded 2. A similar approach was used for liver-related events, counting LT for HPS as a competing event. A cumulative incidence function was calculated to describe the probability of death or LT not for HPS with a 95% CI. Univariate regression analyses were conducted using the Fine and Gray proportional hazards models.

All tests were two-tailed and a *p* value <0.05 was considered significant. Statistical analyses were performed using SPSS version 29.0 software (SPSS Inc, Chicago, IL, USA) and R statistical software version 4.0.2 (R Foundation for Statistical Computing, Vienna, Austria).

## Results

### Prevalence of HPS in patients with PSVD

Out of 313 patients with PSVD and signs of portal hypertension from 17 centers of the French Network for Vascular Liver Diseases, 196 patients with CE-TTE were included into the present study (Fig. S1, Table S2). Patients with and without CE-TTE were similar, except that patients with CE-TTE were younger, had lower serum creatinine and hepatic venous pressure gradient (HVPG), and fewer had ascites than those without CE-TTE at diagnosis of PSVD (Table S3).

CE-TTE was performed as part of the investigations carried out at inclusion in the phase III ‘APIS’ clinical trial (NCT04007289) in 80 patients, as part of the initial work-up performed when patients were referred for PSVD in 94 patients (including 40 who were also included in APIS) and because of dyspnea in 22 patients (including eight who were also included in APIS). Of the 196 patients included in the present study, 128 thus took part in the APIS trial. Out of the 68 patients included in the present study, but not in the APIS trial, reasons for non-inclusion in APIS were refusal to participate in the study (n = 19), strict indication to aspirin (n = 11 including 10 because of myeloproliferative neoplasm), platelet count <40 × 10^9^/L (n = 8), TIPS and/or LT before APIS initiation (n = 7), strict indication to anticoagulation (n = 7), clinically significant active chronic bleeding (n = 6), recent portal vein thrombosis (n = 3), creatinine clearance <30 ml/min (n = 2), malabsorption (n = 2), alcohol intake >140 g/week for women (n = 1), pregnant women (n = 1), and no medical insurance (n = 1).

Out of these 196 patients, 14 (7% [95% CI 3–11]) had a diagnosis of HPS. Hypoxemia (PaO_2_ <80 mmHg) was found in 10 patients with HPS (Patients 2, 3, 5–9, and 12–14), while the other four patients were asymptomatic and had elevated AaPO_2_ without hypoxemia (Patients 1, 4, 10, and 11). HPS was thus diagnosed in 10/22 (45%) symptomatic patients, and in four of 174 (2%) asymptomatic patients. Four (2%) additional patients had intrapulmonary shunts at CE-TTE, but with normal arterial oxygenation (n = 2) or with another cause of hypoxemia (n = 2), so that diagnosis of HPS was not retained. None of the 14 patients with HPS had portopulmonary hypertension, based on right heart catheterization in 13 patients and on echocardiography in one patient (pulmonary artery systolic pressure of 22 mmHg without echocardiographic feature suggestive of portopulmonary hypertension).

Out of the 22 patients with dyspnea as an indication for CE-TTE, causes of respiratory complains were HPS (n = 10), portopulmonary hypertension without (n = 2) or with (n = 1) interstitial lung disease, interstitial lung disease alone (n = 1), obesity (n = 1), anemia (n = 1), granulomatous lymphocytic interstitial lung disease associated with CVID (n = 1), and no cause with spontaneously favorable outcome (n = 5).

### Features associated with HPS in patients with PSVD

Characteristics of patients with PSVD at the time of liver biopsy are presented in Table 1. At the time of PSVD diagnosis, patients with HPS had lower prothrombin index, higher serum alkaline phosphatase (ALK), higher serum total bilirubin, and a lower serum albumin. Moreover, HVPG was higher in patients with HPS than in those without HPS. A total of 132 (68%) patients had at least one extrahepatic condition associated with PSVD, as detailed in Table S4. Genetic disorders associated with PSVD were more frequently observed in patients with HPS than in those without (50% *vs.* 6%, *p* = 0.002). In particular, telomere biology disorders were found in seven (50%) out of the 14 patients with HPS *vs.* six (3%) out of the 182 patients without HPS (*p* <0.001). This was not accounted for by a different rate of genetic testing for these mutations, as telomere biology disorder gene mutations were tested in 10 (71%) patients with HPS *vs.* 100 (55%) patients without HPS (*p* = 0.50). Four of these seven patients with PSVD and HPS and telomere biology disorders have been included in a previous study that reported liver lesions in telomere biology disorders, while the other three were not because they were diagnosed after 2019.^22^ We performed a multivariable analysis including features associated with HPS by univariate analysis, with a value of *p* <0.05, and with <25% of missing variables, namely prothrombin index, serum total bilirubin, and genetic disorder. Genetic disorder was the only variable independently associated with HPS (odds ratio 1.385, 95% CI 1.191–1.610, *p* <0.0001).

**Table 1:** Characteristics of the 196 patients with PSVD at the time of diagnosis of PSVD.

	All patients with PSVD (N = 196)		Patients without HPS (n = 182)		Patients with HPS (n = 14)		*p* value
	n	n (%) or median (IQR)	n	n (%) or median (IQR)	n	n (%) or median (IQR)	
Age, years (range)	187	50 (36–62)	174	50 (38–62)	13	37 (28–57)	0.09
Male sex	196	109 (56)	182	99 (54)	14	10 (71)	0.22
Laboratory data at diagnosis of PSVD							
Leukocytes (G/L)	111	4.3 (3.0–6.0)	104	4.4 (3.1–6.0)	7	4.0 (2.3–4.7)	0.39
Hemoglobin (g/dl)	127	12.7 (11.3–14.0)	119	12.6 (11.4–14)	8	13.3 (10.6–14.0)	>0.9
Mean corpuscular volume (fL)	89	86 (81–91)	84	86 (81–91)	5	93 (92–104)	**0.026**
Platelet count (×10^9^/L)	155	106 (72–161)	145	110 (72–162)	10	88 (31–103)	0.057
Prothrombin index (%)	151	85 (69–97)	142	87 (69–99)	9	69 (55–80)	**0.026**
International normalized ratio	142	1.10 (1–1.22)	132	1.08 (1–1.2)	8	1.16 (1.1–1.39)	0.054
Serum AST (IU/L)	149	35 (25–46)	140	34 (20–43.5)	9	38 (33–48)	0.22
Serum ALT (IU/L)	150	32 (20–45)	141	32 (20–43.5)	9	25 (21–46)	0.91
Serum ALP (IU/L)	136	103 (68–146)	127	101 (68–143)	9	142 (99–343)	**0.048**
Serum GGT (IU/L)	146	66 (32–132)	138	69 (32–132)	8	58 (35–174)	0.83
Serum total bilirubin (μmol/L)	149	13 (9–21)	141	13 (9–19)	8	34 (24–44)	**0.003**
Serum creatinine (μmol/L)	147	70 (60–82)	138	70 (61–82)	9	64 (47–97)	0.30
Serum albumin (g/L)	132	38 (35–42)	126	38 (35–42)	6	35 (33–36)	**0.027**
History or current ascites	193	9 (5)	179	9 (5)	14	0 (0)	>0.9
History or current hepatic encephalopathy	193	1 (1)	179	0 (0)	14	1 (7)	0.07
Vibration-controlled transient elastography							
Liver stiffness (kPa)	108	7.5 (6.1–11.2)	104	7.4 (6.1–11.2)	4	9.6 (8.0–11.5)	0.40
HVPG (mmHg)	104	6 (3–9)	96	6 (3–9)	8	10 (6–16)	**0.039**
Intrahepatic veno-venous collaterals at HVPG measurement	65	31 (48)	63	30 (48)	2	1 (50)	1
Esophageal or gastric varices	130	93 (72)	119	85 (71)	11	8 (73)	>0.9
At least one extrahepatic condition associated with PSVD	193	132 (68)	179	122 (68)	14	10 (71)	1
Immunological disorder	194	67 (35)	180	63 (35)	14	4 (29)	0.77
HIV infection	193	20 (10)	179	20 (11)	14	0 (0)	0.37
Medication or toxin	193	29 (15)	179	29 (16)	14	0 (0)	0.13
Hematological disease and prothrombotic condition	194	45 (23)	180	42 (23)	14	3 (21)	1
Genetic disorder	193	17 (9)	179	10 (6)	14	7 (50)	**<0.001**
At least one other cause of chronic liver disease	196	116 (59)	182	110 (60)	14	6 (43)	0.20
History of excessive alcohol consumption	195	13 (7)	181	12 (7)	14	1 (7)	1
Metabolic comorbidities∗	196	106 (54)	182	100 (55)	14	6 (43)	0.38
Positive anti-HCV antibodies	194	2 (1)	180	2 (1)	14	0 (0)	1
Positive HBs antigen	194	4 (2)	180	4 (2)	14	0 (0)	1
Positive serologic testing for antischistosome antibody	196	9 (5)	182	9 (5)	14	0 (0)	1

Data are presented as median (interquartile range) or n (%) as appropriate. Comparisons of quantitative and qualitative variables were made using the Mann–Whitney *U* test and Χ^2^ or Fisher’s exact tests, respectively. Bolded values indicate statistically significant differences (*p* <0.05). Extrahepatic conditions associated with PSVD are detailed in Table S3.

ALP, alkaline phosphatase; ALT, alanine aminotransferase; AST, aspartate aminotransferase; BMI, body mass index; GGT, gamma-glutamyl transpeptidase; HB, hepatitis B; HCV, hepatitis C virus; HIV, human immunodeficiency virus infection; HPS, hepatopulmonary syndrome; HVPG, hepatic venous pressure gradient; PSVD, porto-sinusoidal vascular disorder.

∗ Metabolic comorbidities included overweight (BMI ≥25 kg/m^2^), diabetes mellitus, arterial hypertension, and/or dyslipidemia.

Nine (5%) of the 196 patients with PSVD had pulmonary fibrosis: three with HPS (Patients 3, 11, and 13) and six without HPS. Two patients with HPS and pulmonary fibrosis had a telomere biology disorder. Among patients with HPS, the three patients with pulmonary fibrosis were similar to those without, except for a higher hemoglobin level (*p* = 0.04) (Table S5). One of these three patients underwent LT, because of HPS requiring long-term oxygen therapy (LTOT) (Patient 3), and then liver retransplantation because of the recurrence of PSVD and HPS requiring LTOT. One patient died of unknown cause (Patient 13). One patient was alive without LTOT (Patient 11).

Characteristics of the patients with PSVD at the time of CE-TTE are presented in Table 2. At CE-TTE, 175 (89%) patients had at least one sign specific for portal hypertension, whereas 21 (11%) only had signs non-specific for portal hypertension. Although the median duration between PSVD diagnosis and CE-TTE was not different between patients with and without HPS, patients with HPS displayed features suggesting a less preserved liver function, namely lower prothrombin index, higher total serum bilirubin, and lower serum albumin. Conversely, no significant difference was observed in patients without and with HPS concerning signs of portal hypertension including portosystemic shunts (66% *vs*. 71%, *p* = 0.77), liver and spleen stiffness, or complications of PSVD. A total of 112 liver biopsies were centrally reviewed by an expert pathologist, including 103 and nine from patients without and with HPS, respectively. As shown in Table S6, HPS tended to be associated with more portal venule obliterations (*p =* 0.085) and with nodular liver architecture (*p =* 0.069).

**Table 2:** Characteristics of patients with PSVD at the time of CE-TTE (diagnosis of HPS).

	All patients with PSVD (N = 196)		Patients without HPS (n = 182)		Patients with HPS (n = 14)		*p* value
	n	n (%) or median (IQR)	n	n (%) or median (IQR)	n	n (%) or median (IQR)	
Age, years (range)	196	55 (42–65)	182	56 (43–65)	14	45 (35–65)	0.15
Duration between diagnosis of PSVD and first CE-TTE (months)	176	17 (2–61)	163	17 (2–60)	13	31 (0–122)	0.73
Body mass index (kg/m^2^)	190	23.61 (21.8–27.1)	176	23.67 (21.9–27)	14	23.02 (20.8–27.7)	0.56
Current or past smoking	194	55 (28)	180	48 (27)	14	7 (50)	0.07
Laboratory data							
Hemoglobin (g/dl)	176	12.9 (11.4–14)	164	12.9 (11.5–14)	12	12.1 (10.1–14.1)	0.50
Platelet count (×10^9^/L)	183	105 (69–165)	170	105 (69–167)	13	101 (68–130)	0.57
Prothrombin index (%)	175	85 (69–100)	162	86 (70–102)	13	63 (61–85)	**0.04**
INR	177	1.1 (1–1.24)	164	1.09 (0.99–1.21)	13	1.26 (1.1–1.3)	0.06
Serum AST (IU/L)	182	38 (29–52)	169	38 (29–52)	13	44 (33–88)	0.1
Serum ALT (IU/L)	185	31 (21–47)	172	31 (21–47)	13	22 (18–46)	0.21
Serum ALP (IU/L)	154	112 (74–175)	141	104 (70–159)	13	181 (135–286)	**0.004**
Serum GGT (IU/L)	184	70 (32–132)	171	69 (32–131)	13	73 (36–169)	0.66
Serum total bilirubin (μmol/L)	185	15 (10–24)	172	14 (10–21)	13	37 (23–65)	**<0.001**
Serum creatinine (μmol/L)	170	70 (60–86)	157	70 (61–86)	13	59 (46–83)	**0.048**
Serum albumin (g/L)	170	38 (35–41)	160	38 (35–41)	10	32 (24–34)	**<0.001**
Signs of portal hypertension							
Thrombocytopenia	195	138 (71)	181	135 (69)	13	13 (93)	0.07
Ascites	196	16 (8)	182	15 (8)	14	1 (7)	1
Splenomegaly	191	147 (77)	177	135 (76)	14	12 (86)	0.53
Portosystemic collaterals at imaging	192	142 (74)	178	129 (73)	14	13 (93)	0.12
Small esophageal varices	186	64 (34)	173	62 (36)	13	2 (15)	0.23
Gastric or large esophageal varices, or history of variceal band ligation	191	107 (56)	177	98 (55)	14	9 (64)	0.52
Vibration-controlled transient elastography							
Liver stiffness (kPa)	167	7.4 (5.9–10.8)	160	7.35 (5.8–10.2)	7	11.7 (6.1–24.6)	0.09
Spleen stiffness (kPa)	95	49.4 (30.8–74.7)	90	48.2 (30.7–75)	5	54.1 (31.6–55.3)	0.71
Complications of PSVD until CE-TTE							
History of ascites	195	27 (14)	181	25 (14)	14	2 (14)	1
History of hepatic encephalopathy	195	6 (3)	181	4 (2)	14	2 (14)	0.06
History of esophageal or gastric variceal bleeding	196	28 (14)	182	27 (15)	14	1 (7)	0.7
History of portal vein and/or left or right branches thrombosis	196	40 (20)	182	38 (21)	14	2 (14)	0.74
History of mesenteric venous and/or splenic vein thrombosis	196	10 (5)	182	9 (5)	14	1 (7)	0.53
Medications							
Anticoagulation therapy	164	24 (15)	150	23 (15)	14	1 (7)	0.7
Diuretic therapy	163	20 (12)	149	17 (11)	14	3 (21)	0.38
Non-selective β-blockers	164	69 (42)	150	63 (42)	14	6 (43)	0.95
Cardio-selective β-blockers	164	4 (2)	150	4 (3)	14	0 (0)	1

Data are presented as median (IQR) or n (%) as appropriate. Comparisons of quantitative and qualitative variables were made using the Mann–Whitney *U* test and Χ^2^ or Fisher’s exact tests, respectively. Bolded values indicate statistically significant differences (*p* <0.05).

ALP, alkaline phosphatase; ALT, alanine aminotransferase; AST, aspartate aminotransferase; CE-TTE, contrast-enhanced transthoracic echocardiography; GGT, gamma-glutamyl transpeptidase; HPS, hepatopulmonary syndrome; INR, international normalized ratio; PSVD, porto-sinusoidal vascular disorder.

**Fig. 1:**
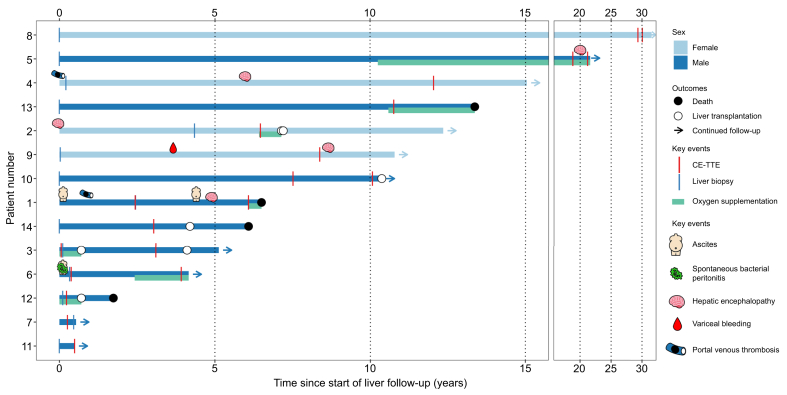
Outcome of the 14 patients with PSVD and HPS. CE-TTE, contrast-enhanced transthoracic echocardiography; HPS, hepatopulmonary syndrome; PSVD, porto-sinusoidal vascular disorder.

Detailed features of the 14 patients with PSVD and HPS at the time of liver biopsy and at the time of CE-TTE are presented in Table 3, Table 4. Three (21%) patients had severe HPS (Patients 3, 6, and 12). A total of six (43%) patients required LTOT and one more patient needed oxygen therapy for exercise (Patient 13). Detailed respiratory features at the time of CE-TTE and outcome of the 14 patients with PSVD and HPS are presented in Table 5 and Fig. 1.

**Table 3:** Characteristics of patients with PSVD and HPS at the time of liver biopsy (diagnosis of PSVD) (n = 14).

Patient	Sex	Age (years)	HVPG (mmHg)	Extrahepatic condition associated with PSVD	Other cause of chronic liver disease
1	Male	67	5	None	Metabolic comorbidities (overweight, arterial hypertension, diabetes) and past excessive alcohol consumption
2	Female	63	Not performed	Sjögren’s syndrome	None
3	Male	34	12	TBD (*TERT* mutation)	None
4	Female	23	7	None	None
5	Male	30	5	TBD (*TERT* mutation)	None
6	Male	38	14	CVID	None
7	Male	22	8	TBD (*TERC* mutation)	None
8	Female	2	Not performed	None	None
9	Female	64	16	CVID	Metabolic comorbidities (arterial hypertension)
10	Male	35	Not performed	TBD, Heterozygous factor V Leiden mutation	None
11	Male	55	Not performed	None	Metabolic comorbidities (arterial hypertension, dyslipidemia)
12	Male	47	17	TBD (*DKC1* mutation), diffuse large B cell lymphoma complicated by immune deficiency	None
13	Male	54	Not performed	TBD (*TERT* mutation)	None
14	Male	33	Not performed	TBD (*TERC* mutation) and myelodysplastic syndrome	Metabolic comorbidities (overweight)

CVID, common variable immune deficiency; HPS, hepatopulmonary syndrome; HVPG, hepatic venous pressure gradient; PSVD, porto-sinusoidal vascular disorder; TBD, telomere biology disorder.

**Table 4:** Characteristics of patients with PSVD and HPS at the time of CE-TTE (diagnosis of HPS) (n = 14).

Patient	Age (years)	BMI (kg/m^2^)	Current or past smoking (pack-year)	INR	Serum total bilirubin (μmol/L)	Serum creatinine (μmol/L)	Specific signs of portal hypertension	Non-specific signs of portal hypertension	Liver stiffness (kPa)	Complications of PSVD
1	67	29.4	Past (40)	1.39	44	125	Portosystemic collaterals at imaging, history of band ligation, persistent large EV and GV	Thrombocytopenia, splenomegaly, history of ascites	8.3	History of ascites, history of portal thrombosis
2	65	21.9	No	1.27	126	40	Portosystemic collaterals at imaging	Thrombocytopenia, splenomegaly		Hepatic encephalopathy
3	34	20.4	Current (17)	1.10	32	52	Portosystemic collaterals at imaging, large EV	Thrombocytopenia, splenomegaly		
4	35	22.6	No	1.26	51	63	Portosystemic collaterals at imaging, history of band ligation, persistent large EV	Thrombocytopenia, splenomegaly		History of hepatic encephalopathy and portal thrombosis
5	49	20.9	Past (30)	1.14	22	36	Portosystemic collaterals at imaging	Thrombocytopenia, splenomegaly	11.7	
6	38	14.6	Past	1.11	37	17	None	History of ascites		History of ascites and spontaneous bacterial peritonitis
7	22	22.9	No				Portosystemic collaterals at imaging, history of large EV, persistent small EV	Thrombocytopenia, splenomegaly		
8	31	29.4	No	1.37	30	57	Portosystemic collaterals at imaging, large EV	Thrombocytopenia, splenomegaly		
9	73	20.4	Past (35)	1.27	79	51	Portosystemic collaterals at imaging, history of portal hypertensive bleeding	Thrombocytopenia, splenomegaly	24.6	History of portal hypertensive bleeding
10	42	27.2	No	1.35	48	59	Portosystemic collaterals at imaging, history of large EV, persistent small EV and GV	Thrombocytopenia, splenomegaly	21.5	
11	56	24.6	No	1.10	8	85	Portosystemic collaterals at imaging	Thrombocytopenia, splenomegaly	6.1	
12	47	24.9	Past (15)	0.87	16	106	Portosystemic collaterals at imaging	Thrombocytopenia, splenomegaly	61	
13	65	23.2	Past (1)	1.31	24	81	Portosystemic collaterals at imaging, large EV	Thrombocytopenia	5	
14	36	29.8	No	1.08	151	61	Portosystemic collaterals at imaging, large EV	Thrombocytopenia, splenomegaly		Jaundice

BMI, body mass index; CE-TTE, contrast-enhanced transthoracic echocardiography; EV, esophageal varices; GV, gastric varices; HPS, hepatopulmonary syndrome; INR, international normalized ratio; PSVD, porto-sinusoidal vascular disorder.

**Table 5:** Respiratory features at the time of CE-TTE, and outcomes of patients with PSVD and HPS after CE-TTE (n = 14).

Patient	Number of cardiac cycles for bubbles detection	Arterial oxygen saturation (%)	Duration between CE-TTE and arterial blood gases (months)	PaO_2_ (mmHg)∗		AaPO_2_ (mmHg)		Outcome of HPS	Complications of PSVD after CE-TTE	Other major complications
				In sitting position	In supine position	In sitting position	In supine position			
1	5		32	81		24		LTOT	Hepatic encephalopathy, ascites	Death from liver-related cause 6 months after HPS diagnosis
2	5		6	68 (LTOT 3 L/min)	149	32	65	LTOT, LT	—	Re-LT for ischemic cholangitis, PML after LT
3			0	57		54		LTOT, LT	—	Re-LT for recurrence of PSVD and HPS
4	5	97	0	84		26		Asymptomatic	—	
5	4	98	0	78		30		LTOT	Hepatic encephalopathy,	
6	6		0	120 (FiO_2_ 100%)	180 (FiO_2_ 100%)			LTOT	Ascites	
7			0	61	61	50	51	Dyspnea		
8	3	95	11	77	83	29	23	Dyspnea	—	
9	5	97	0	77		35		Dyspnea	Hepatic encephalopathy	
10	5	98	13	87		15		Asymptomatic, LT	Hepatic encephalopathy, jaundice, HCC on the explant	
11	5	98	0	88		15		Asymptomatic	—	
12	5	94	0	56	66	59	42	LTOT, LT	—	Death from recurrence of PSVD and HPS with LTOT
13	5		0	79		18		Oxygen therapy for exercise	—	Death (cause unknown)
14	5		3	66	70	42		LT	—	Death from hematological disease

AaPO_2_, alveolar–arterial oxygen gradient; CE-TTE, contrast-enhanced transthoracic echocardiography; EV, esophageal varices; FiO_2_: Fraction of inspired oxygen; GV, gastric varices; HCC, hepatocellular carcinoma; HPS, hepatopulmonary syndrome; LT, liver transplantation; LTOT, long-term oxygen therapy; PaO_2_, partial pressure of arterial oxygen; PML, progressive multifocal leukoencephalopathy; PSVD, porto-sinusoidal vascular disorder; Re-LT, liver retransplantation.

∗ Hypoxemia was defined as a PaO_2_ <80 mmHg.

Out of the 14 patients with HPS, the 10 patients with respiratory symptoms more frequently had an extrahepatic condition associated with PSVD (*p =* 0.041) and a higher A–a oxygen gradient at diagnosis of HPS than those without symptoms (*p =* 0.017) (Table S7).

The four patients with intrapulmonary shunts at CE-TTE, but without HPS, reported no dyspnea during a median follow-up of 15 (range 10–55) months.

### Patient outcomes

The 196 patients with PSVD had a median follow-up time of 23 (6–36) months from CE-TTE to end of follow-up. During that period of time, onset or worsening of ascites occurred in 11 (6%) patients, spontaneous bacterial peritonitis in three (2%) patients, onset or worsening of hepatic encephalopathy in 10 (6%) patients, gastrointestinal bleeding caused by portal hypertension in 11 (6%) patients, and development or extension of portal thrombosis in 16 (9%) patients. One patient had a diagnosis of hepatocellular carcinoma (on the explant after LT). Two (1%) patients required a TIPS, one for gastrointestinal bleeding caused by portal hypertension not controlled by medical or endoscopic treatment (patient without HPS) and another for portal vein thrombosis 15 years after PSVD diagnosis (Patient 4 with HPS), 10 (5%) underwent LT, and 16 (9%) patients died, including four liver-related deaths. Follow-up duration after CE-TTE was longer in patients with HPS than in those without (34 months [24–46] *vs.* 22 months [6–32], *p* = 0.02).

Out of the 14 patients with HPS, five (36%) underwent LT, because of HPS requiring LTOT for three of them (Patients 2, 3, and 12), and because of liver failure for two patients (Patients 10 and 14). Histological analysis of liver explants confirmed PSVD without cirrhosis in all five cases of patients with HPS who underwent LT. In one patient, two nodules of hepatocellular carcinoma were diagnosed on the explant (Patient 10). No recurrence of hepatocellular carcinoma occurred after a follow-up of 10 months. Out of the three patients who underwent LT because of HPS requiring LTOT, HPS improved after LT in one patient (Patient 2), whereas HPS recurred after LT in the other two patients requiring further LTOT (Patients 3 and 12). Two patients underwent liver retransplantation – one because of ischemic cholangitis (Patient 2) and one because of recurrence of PSVD and HPS requiring further LTOT (Patient 3). Both were alive 62 and 12 months after the last LT, respectively, one without dyspnea or need for oxygen therapy (Patient 2) and the other with a new recurrence of PSVD and of HPS after the second transplantation (Patient 3). Two patients died after LT – one as a result of the recurrence of PSVD shortly after LT accompanied with recurrence of HPS requiring LTOT (Patient 12), and one from a hematological disease (Patient 14). Two additional patients with HPS died, one owing to a liver-related cause and one of unknown cause.

**Fig. 2:**
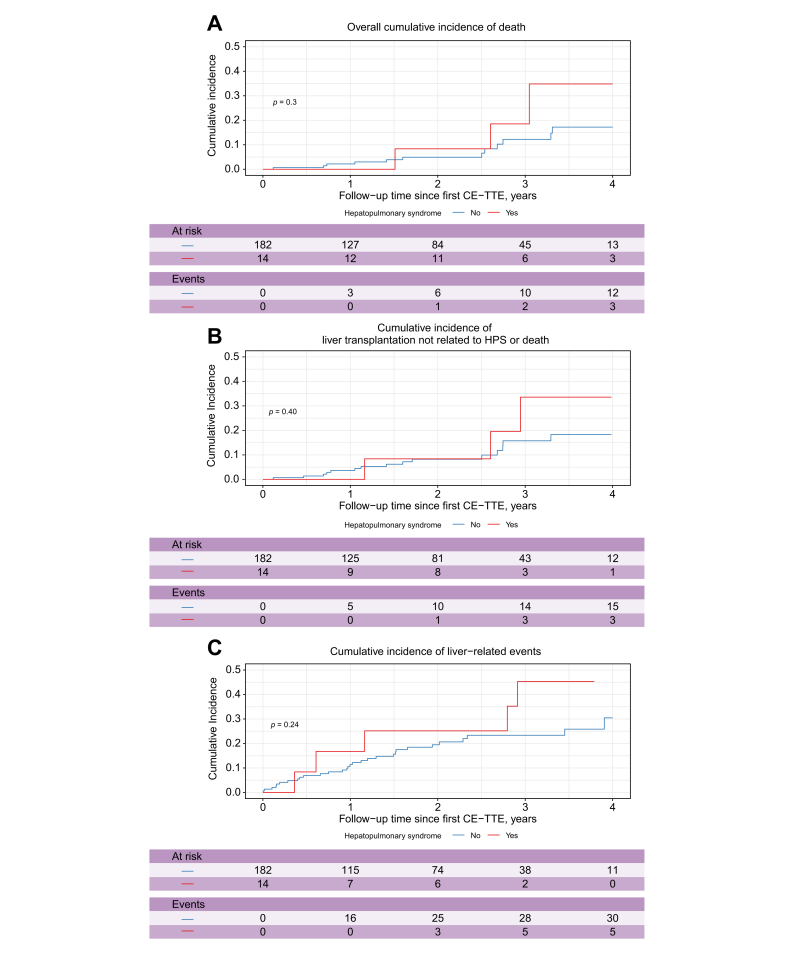
Outcome of patients with PSVD, from the date of CE-TTE, according to presence or not of HPS. (A) Overall cumulative incidence of death. Overall cumulative incidence of death from the date of CE-TTE was assessed using the Kaplan–Meier method and comparison between patients with and without HPS was performed using the log-rank test. (B) Cumulative incidence of LT not related to HPS or death (LT for HPS considered as a competing risk). The Fine and Gray model was used and comparison between patients with and without HPS was performed using Gray’s test. (C) Cumulative incidence of the liver-related events (*i.e.* as onset or worsening of ascites, spontaneous bacterial peritonitis, onset or worsening of hepatic encephalopathy, gastrointestinal bleeding caused by portal hypertension, or portal venous thrombosis), or LT not related to HPS, or liver-related death (LT for HPS considered as a competing risk). The Fine and Gray model was used and comparison between patients with and without HPS was performed using Gray’s test. CE-TTE, contrast-enhanced transthoracic echocardiography; HPS, hepatopulmonary syndrome; PSVD, porto-sinusoidal vascular disorder.

Overall cumulative incidence of death was similar between patients with and without HPS (Fig. 2A). LT was more commonly performed in patients with than in those without HPS (36% *vs.* 3%; *p* = 0.003 and Fig. S2). As HPS is indication for LT, we performed competing risk analyses considering LT for HPS as a competing risk. We observed that patients with HPS had a cumulative incidence of liver-related events and of death or LT not for HPS similar to that of patients without HPS (Fig. 2B and C). Features at CE-TTE associated with patients’ outcome are presented in Tables S8 and S9.

When focusing on the four patients with PSVD and HPS but without respiratory symptoms at HPS diagnosis, Patient 1 became symptomatic and required LTOT, Patient 10 underwent LT because of liver failure, and Patient 4 remained asymptomatic after 36 months of follow-up. No follow-up data were available for Patient 11. Cumulative incidence of death and of death or LT between the 182 patients without HPS and the four patients with asymptomatic HPS at diagnosis were similar (*p =* 0.5 and *p =* 0.6, respectively).

### Plasma concentrations of angiogenic and inflammatory mediators and of endotoxin in HPS

**Fig. 3:**
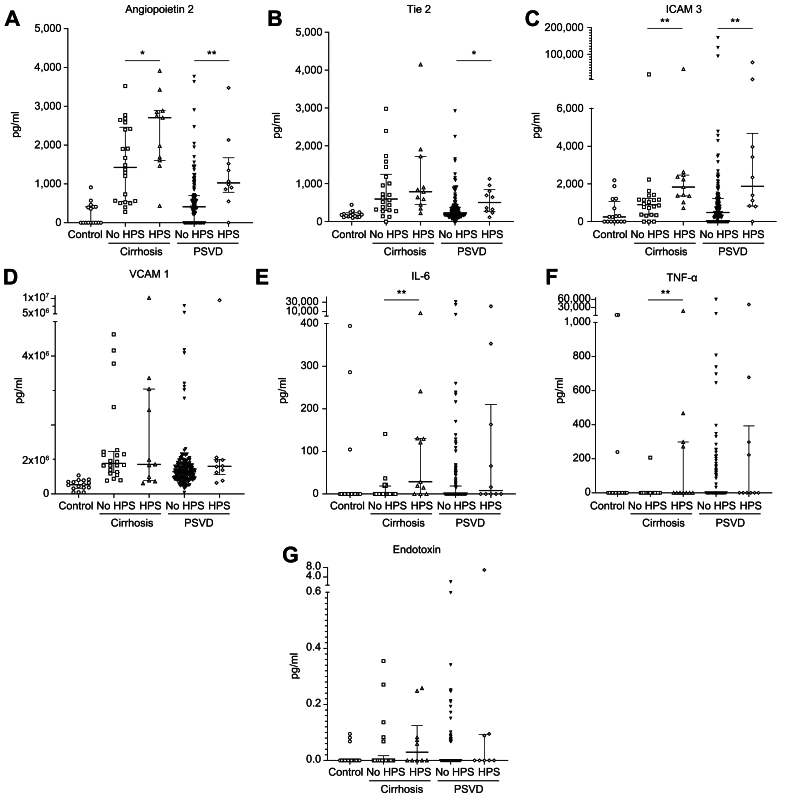
Plasma concentrations of angiogenic and inflammatory mediators and of endotoxin in patients with and without HPS according to liver disease (PSVD and cirrhosis) and in healthy individuals. (A) Angiopoietin 2. (B) Tie2. (C) ICAM 3. (D) VCAM1. (E) IL-6. (F) TNF-α. G. Endotoxin. Scatterplots representing the concentrations with median and interquartile range. To avoid multiple testing, we only performed statistical analyses for comparisons between patients without and with HPS; ∗*p* value <0.05; **∗∗***p* value <0.01 (Mann–Whitney *U* test). HPS, hepatopulmonary syndrome; ICAM3, intercellular adhesion molecule 3; IL-6, interleukin 6; PSVD, porto-sinusoidal vascular disorder; Tie2, TEK tyrosine kinase endothelial; TNF-α, tissue necrosis factor alpha; VCAM1, vascular cell adhesion molecule 1.

Out of the 196 patients with PSVD, plasma was available for measurement of angiogenic and inflammatory mediators in 161 patients, including 151 without HPS and 10 with HPS. As a reference, these mediators were also measured in 33 patients with cirrhosis, including 22 without and 11 with HPS and in 16 healthy individuals (nine women; median age 36, IQR 30–46). Characteristics of patients with cirrhosis at the time of plasma collection are presented in Table S10. Median MELD was 13;[10], [11], [12], [13], [14], [15], [16], [17] Child–Pugh score was A, B, and C in 10, 16, and seven patients, respectively. Plasma concentrations of angiogenic and inflammatory mediators are shown in Fig. 3. To avoid multiple testing, we only performed statistical analyses for comparisons between patients without and with HPS. In both PSVD and cirrhosis, patients with HPS had higher plasma concentrations of ICAM3 and Angiopoietin 2 than those without HPS. Plasma Tie2 concentrations were also higher in patients with PSVD and HPS than in those with PSVD without HPS, but this difference was not observed in cirrhosis. Patients with cirrhosis and HPS had higher plasma concentrations of TNF-α and IL-6 than those without HPS, but this difference was not observed in patients with PSVD. HPS was not associated with plasma concentrations of VCAM1 or endotoxin in either PSVD or cirrhosis.

## Discussion

Despite the rarity of PSVD, the present retrospective multicenter study was able to include a large cohort of patients screened for HPS. We found a prevalence of HPS of 7% in PSVD and that HPS was associated with genetic disorders as well as with features suggesting a less preserved liver function. HPS in PSVD was associated with higher plasma concentration of angiogenic mediators, but not of inflammatory mediators.

The first finding of the present study was a prevalence of HPS of 7% (95% CI 3–11%) in patients with PSVD. These results are consistent with those described in two previous studies gathering 19 and 24 PSVD patients, with an estimated prevalence of HPS of 8% (95% CI 0–19%) and 11% (95% CI 0–24%).^16^^,^^17^ This prevalence is in the range of that described in patients with cirrhosis, varying between 4% and 32%.[23], [24], [25], [26], [27] Still, this caution is needed for the following reasons: (i) the entire population was not screened (109 patients were excluded because the CE-TTE was unperformed); (ii) among the 17 participating centers, 13 included fewer than five patients and eight included only one patient, possibly leading to a selection bias; (iii) screening was conducted at different times of the natural history of the disease and for various indications. Therefore, a prospective validation of our findings with extension to other international centers would be useful in the future. In practice, our results suggest that all patients with dyspnea should be screened for HPS, as almost half of them had HPS. In a recent VALDIG study of LT for PSVD, HPS was the third cause of LT, reinforcing the idea of screening symptomatic patients.^28^ In those without respiratory symptoms, the prevalence of HPS is low (2%). Moreover, patients with asymptomatic HPS have a similar outcome to those without HPS, so there is probably no harm in waiting for symptoms to develop to screen for HPS. However, this view should be treated with caution as it is based on only four patients with HPS without respiratory symptoms.

The second major finding of this study is the identification of features associated with HPS in PSVD, which had not been possible so far because of the limited sample size of previous studies (Table S1). We observed here that HPS was associated with genetic disorders, especially telomere biology disorders. This finding is in line with two previous studies: one recent study showing that PSVD is the most frequent liver lesion found in patients with telomere biology disorders,^22^ and another reporting that HPS is frequent in patients with telomere syndrome.^29^ This particular association between telomere biology disorders and HPS, together with other vascular abnormalities common in patients with telomere biology disorders, namely gastrointestinal telangiectatic anomalies, pulmonary arteriovenous malformations, and retinal vascular anomalies, suggests that extrahepatic endothelial senescence may contribute to HPS in addition to the liver disease itself.^30^ We also observed that patients with HPS had a less preserved liver function, namely lower prothrombin index, higher total serum bilirubin, and lower serum albumin. This could reflect more long-standing liver disease in patients with HPS, as could the association of HPS with genetic disorders, where it can be assumed that liver disease begins early in life. This association between HPS and impaired liver function seems to have some specificity for PSVD as such a link is usually not observed in patients with cirrhosis.^31^ Likewise, HPS tended to be associated with more pronounced histological lesions of PSVD, namely more portal venule obliterations and more nodular liver architecture, although significance was not reached possibly because of a lack of power. Conversely, HPS was not associated with any feature reflecting portal hypertension. HVPG was slightly higher in patients with HPS than in those without in the subgroup of patients in whom this measurement was available, but HVPG in PSVD is not a reliable reflection of portal hypertension because of a presinusoidal block.^32^

The third major finding of our study was the outcome of patients with HPS in PSVD. We observed no difference in overall cumulative incidence of death between patients without and with HPS, most likely thanks to the HPS MELD exception policy for LT that was applied. When considering LT for HPS as a competing risk, we also found no difference in the cumulative incidence of LT or death nor of liver-related events. This suggests that the higher rate of LT performed in patients with HPS allowed survival of patients with HPS up to the level of that of patients without HPS, as reported in patients with cirrhosis.[33], [34], [35] Limitations of our analyses are that the baseline for the survival analyses was CE-TTE, which was performed at different times in the disease course and for different reasons, and that some patients without HPS at baseline may have developed HPS during follow-up, which is quite long (median 23 months, IQR 6–36). Furthermore, the similar overall survival of patients with and without HPS should not mask the significant morbidity associated with HPS in the pre- and post-transplant setting: out of the three patients who underwent LT because of HPS requiring LTOT, two patients required a second transplantation (one because of HPS recurrence, the other because of ischemic cholangitis) and another died post-transplant as a result of HPS recurrence. Future studies should investigate quality of life in patients with PSVD and HPS, as has been done in cirrhosis.^9^

Finally, our study leveraged the large biobanking performed in our network to provide insights into potential mechanisms involved in the pathogenesis of HPS in PSVD. The current view of the pathophysiology of HPS, mainly derived from experiments conducted in animals after bile duct ligation and with cirrhosis, points to bacterial translocation with pulmonary intravascular recruitment of immune cells, pulmonary endothelial dysfunction, angiogenesis, and AT2 cell dysfunction as the most important mechanisms.^9^^,^^10^ We therefore investigated plasma concentrations of angiogenic and inflammatory markers and of endotoxin in patients with PSVD and cirrhosis. We demonstrated that HPS in PSVD is characterized by high plasma concentration of angiogenic mediators (Angiopoietin 2, ICAM3, and Tie2), similarly to what happens in cirrhosis.^11^ Conversely to the cirrhosis setting, no difference was observed in plasma inflammatory markers (TNF-α and IL-6). This suggests either that the pathophysiology of HPS in PSVD might not be identical to that in cirrhosis, or that lung inflammatory changes associated with HPS are not reflected by plasma concentrations of inflammatory mediators. However, the markers we have selected provide a limited view of the complex biological processes involved and are unlikely to capture the full range of underlying mechanisms. Recent advances in single-cell RNA sequencing and spatial transcriptomics offer a more unbiased and detailed exploration of cellular and molecular mechanisms. Future studies could incorporate these technologies to more comprehensively investigate the pathophysiology of HPS in PSVD.

In conclusion, this study demonstrates that the prevalence of HPS in PSVD is in the range of that observed in patients with cirrhosis. Genetic disorders, and especially telomere biology disorders, are particularly associated with HPS in this patient population. Angiopoietin 2, Tie 2, ICAM3 levels were associated with HPS in patients with PSVD, whereas TNF-α and IL-6 and endotoxin were not. When applying HPS MELD exception policy for LT, overall survival of patients with PSVD and HPS was similar to that of patients with PSVD without HPS.

## Abbreviations

A–a gradient, alveolar–arterial gradient; AaPO_2,_ alveolar–arterial oxygen gradient; AT2, alveolar type II; CE-TTE, contrast-enhanced transthoracic echocardiography; CPP, Committee for the Protection of Persons Concerned; CT, computed tomography; CVID, common variable immune deficiency; EV, esophageal varices; FiO_2_, fraction of inspired oxygen; GGT, gamma-glutamyl transferase; GV, gastric varices; HCC, hepatocellular carcinoma; HPS, hepatopulmonary syndrome; HVPG, hepatic venous pressure gradient; ICAM3, intercellular adhesion molecule 3; INR, international normalized ratio; LT, liver transplantation; LTOT, long-term oxygen therapy; MELD, model for end-stage liver disease; MRI, magnetic resonance imaging; PaO_2_, partial pressure of arterial oxygen; PML, progressive multifocal leukoencephalopathy; PSVD, porto-sinusoidal vascular disorder; Re-LT, liver retransplantation; TBD, telomere biology disorder; Tie2, TEK tyrosine kinase endothelial; TNF-α, tissue necrosis factor alpha; TIPS, transjugular intrahepatic portosystemic shunt; VALDIG, Vascular Liver Disease Interest Group; VCAM1, vascular cell adhesion molecule 1.

## Financial support

Funding source: P-ER’s laboratory receives financial support from the Fondation pour la Recherche Médicale (FRM EQU202303016287), “Institut National de la Santé et de la Recherche Médicale” (ATIP AVENIR), the “Agence Nationale pour la Recherche” (ANR-18-CE14-0006-01, RHU QUID-NASH, ANR-18-IDEX-0001, and ANR-22-CE14-0002) by ‘Émergence, Ville de Paris’, by Fondation ARC, by the European Union’s Horizon 2020 research and innovation programme under grant agreement No. 847949 (DECISION) and No. 825575 (RiTa), and by France 2030 RHU LIVER-TRACK (ANR-23-RHUS-0014). The sponsor of the APIS trial was *Assistance Publique – Hôpitaux de Paris* (Clinical Research and Development Department). The APIS trial was funded by a grant from Programme Hospitalier de Recherche Clinique - PHRC 2017 (Ministry of Health).

## Authors’ contributions

Conceptualization and methodology: PER (lead), SS, YS. Data curation: YS. Formal analysis: SS (lead), KEH, YS, PER. Funding acquisition: PER (lead). Investigation: SS (lead), YS (lead), KEH, MT, OG, VM, APR, AG, AL, DT, JBN, IH, CC, AH, PHD, SH, NGC, ND, SRV, LM, MT, EM, AP, FD, SR, AC, LE, PER. Project administration: SS, YS, PER (lead). Supervision: PER (lead), SS. Writing – original draft: SS (lead), YS (lead), PER. Writing – review and editing: SS (lead), YS (lead), KEH, MT, OG, VM, APR, AG, AL, DT, JBN, IH, CC, AH, PHD, SH, NGC, ND, SRV, LM, MT, EM, AP, FD, SR, AC, LE, PER (lead).

## Data availability statement

The authors are prepared to provide the data from this study upon request to the corresponding author.

## Conflicts of interest

P-ER has received research funding from Terrafirma and acted as consultant for Hemostod, Mursla, Genfit, Boehringer Ingelheim, and Abbelight, and received speaker fees from Tillots pharma and AbbVie. CC received research funding from Gilead and Ipsen, and speaker fees from AbbVie, Intercept, and Gilead.

Please refer to the accompanying ICMJE disclosure forms for further details.
