# Supplementary material for: Hepatopulmonary syndrome in patients with porto-sinusoidal vascular disorder: Characteristics and outcome
Source: JHEP Rep. 2024 Dec 20;7(4):101310. doi: 10.1016/j.jhepr.2024.101310 (PMC11960633; doi:10.1016/j.jhepr.2024.101310)
Supplement: Multimedia component 1 [file mmc1.pdf]

Hepatopulmonary syndrome in patients with porto-sinusoidal vascular disorder: Characteristics and outcome

Sabrina Sidali, Ylang Spaes, Kinan El Husseini, Odile Goria, Vincent Mallet, Armelle Poujol-Robert, Anne Gervais, Adrien Lannes, Dominique Thabut, Jean-Baptiste Nousbaum, Isabelle Hourmand-Ollivier, Charlotte Costentin, Alexandra Heurgué, Pauline Houssel-Debry, Sophie Hillaire, Nathalie Ganne-Carrié, Nicolas Drilhon, Shanta Ram Valainathan, Lucile Moga, Marion Tanguy, Estelle Marcault, Aurélie Plessier, François Durand, Sarah Raevens, Valérie Paradis, Agnès Cachier, Laure Elkrief, Pierre-Emmanuel Rautou

Table of contents

Supplementary figures……………………………………………………………………...2

Supplementary tables…………………………………………………………………………4

Supplementary references…………………………………………………………………..18

Supplementary figures

Fig. S1. Flow chart of the study.

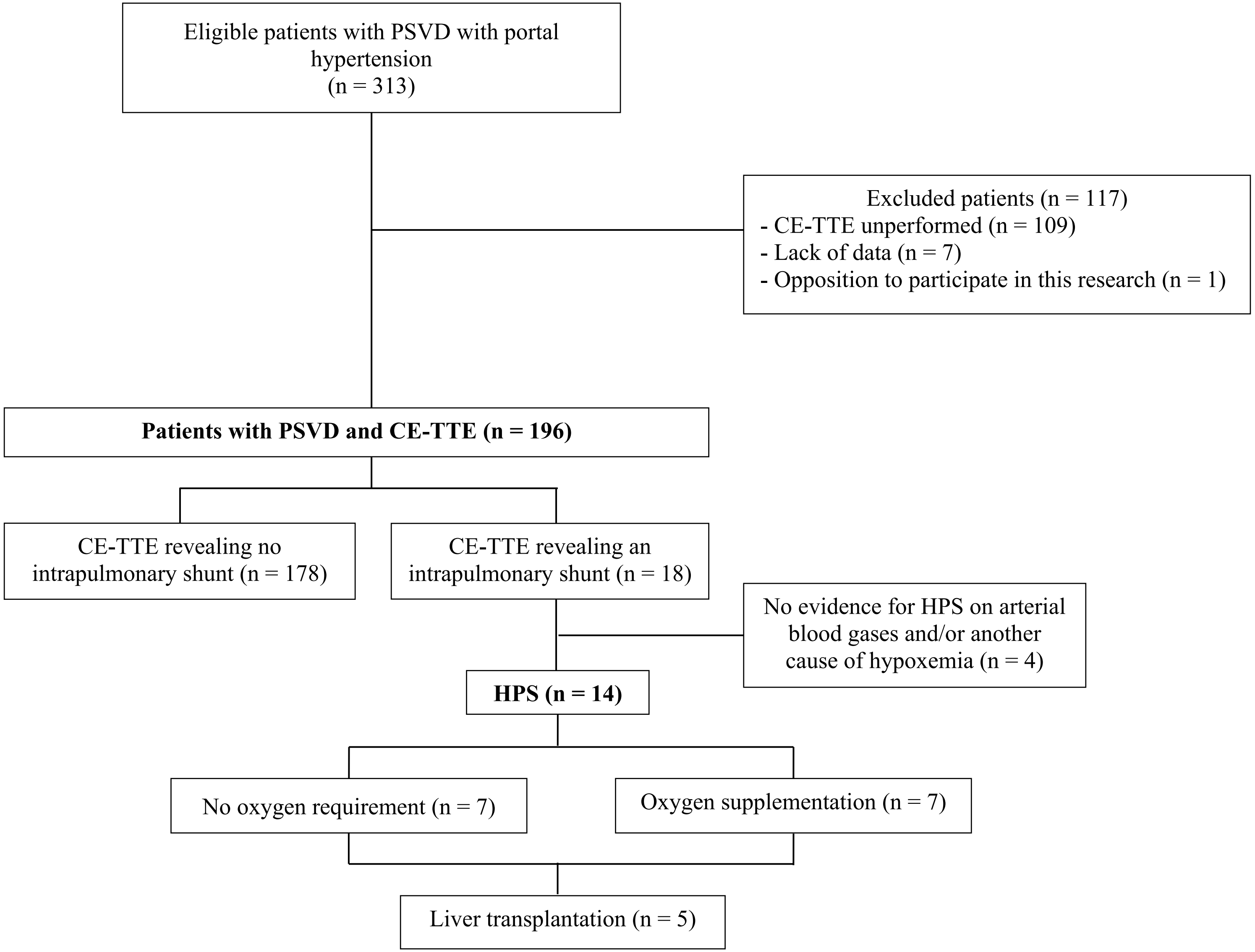

Abbreviations: CE-TTE, contrast-enhanced transthoracic echocardiography; HPS, hepatopulmonary syndrome; PSVD, porto-sinusoidal vascular disorder.

Fig. S2. Overall cumulative incidence of liver transplantation or death in patients with PSVD with and without HPS (Kaplan-Meier curves).

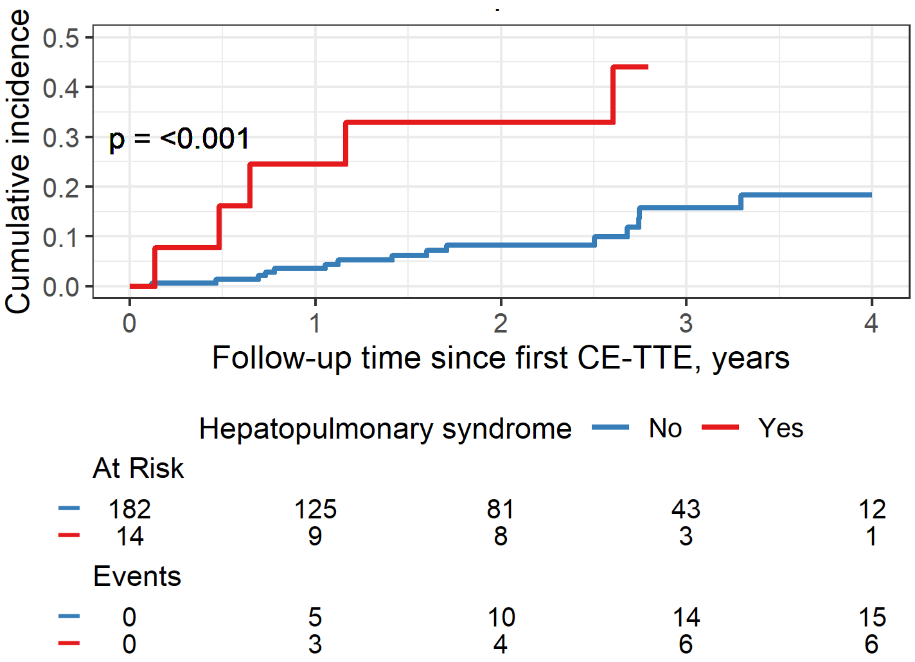

Overall cumulative incidence of liver transplantation or death from the date of CE-TTE was assessed using the Kaplan-Meier method and comparison between patients with and without HPS was performed using the log-rank test.
Abbreviations: CE-TTE, contrast-enhanced transthoracic echocardiography.

Supplementary tables

Table S1. Summary of previous studies describing patients with HPS in context of PSVD.

Author, year	Number of patients	Sex	Identified risk factor of PSVD	HVPG (mmHg)	Age at diagnosis of HPS (years)	Median platelet count at diagnosis of HPS (G/L)	EV at diagnosis of HPS	Arterial oxygenation data				Outcome
								Hypoxemia	Orthodeoxia	PaO_2 (mmHg)	$AaPO_2$ (mmHg)	
Marchand EJ. (1), 1962	4	F	Schistosomiasis	NA	10y	68	No	NA	NA	NA	NA	NA
		F			36y	144	No					NA
		M			54y		Yes					NA
		F			11y	64	Yes					Death at 14 years old
Babbs C. (2), 1988	1	M	NA	7	13y	94	Yes (large)	Yes	NA	57	53	NA
Krowka MJ. (3), 2000	1	M	NA	NA	58y	NA	NA	Yes	Yes	50 in supine position; 33 in sitting position	87 in sitting position; 65 in supine position	Death from hepatocellular carcinoma
Binay K De. (4), 2000	2		NA	NA	NA	NA	NA	NA	Yes	76 in supine position; 68 in standing position	40	Stability at 9 months
									Yes	68 in supine position; 63 in standing position	50	Stability at 7 months
Kaymakoglu S. (5), 2003	2	F	NA	NA	30y	NA	Yes	Yes	Yes	51	69	NA
		M			17y			No		89	23	
Taille C. (6), 2003	1	M	NA	NA	17y	NA	NA	Yes	NA	64	41	LT
Swanson KL. (7), 2005	1	M	Myelodysplastic syndrome	NA	65y	NA	NA	Yes	NA	37	NA	Death from lymphoma
Krasinska AM. (8), 2005	3	NA	NA	NA	NA	NA	NA	NA	NA	NA	NA	LT
Yilmaz S. (9), 2005	1	M	NA	6	18y	83	Yes (large)	Yes	Yes	53 in supine position; 45	68	On liver transplant list

										in sitting position		
Deibert P. (10), 2006	1	M	NA	NA	31y	NA	NA	Yes	NA	36	NA	NA
Gupta S. (11), 2010	3		NA	NA	37y	NA	NA	Yes	NA	49	68	LT
					60y			Yes		48	57	
					30y			Yes		43	71	
Maganty K. (12), 2011	1	F	NA	15	24y	147	Yes	Yes	Yes	42	78	LT for LTOT
Cazals-Hatem D. (13), 2011	2		NA	NA	NA	NA	NA	NA	NA	NA	NA	NA
Naalsund A. (14), 2011	1	M	CVID	16	62y	NA	NA	Yes	Yes	63 in supine position; 48 in sitting position	57 in supine position; 74 in sitting position	LT
Germán Muñoz Maya O., 2012	1	F	Protein C and S deficiency	NA	21y	87	NA	Yes	NA	57	30	LTOT
Cantez MS. (15), 2013	1	NA	NA	NA	NA	NA	NA	NA	NA	NA	NA	LT
Franchi-Abella S. (16), 2014	2	NA	NA	NA	NA	NA	NA	NA	NA	NA	NA	LT
Alhosh R. (17), 2014	1	M	Solid organ transplantation, immunosuppressive treatments	NA	3y	NA	NA	Yes	NA	NA	NA	LT for LTOT
Holmes SN. (18), 2015	2	M	CVID	NA	32y	NA	NA	Yes	No	56	52	NA
		M	CVID	NA	37y	NA	NA	Yes	Yes	66	38	NA
Gorgy AI. (19), 2015	1	M	TBD (*TERT*)	NA	24y	NA	NA	NA	NA	NA	NA	NA
	1	M	TBD (*DKC1*)	NA	34y	NA	Yes	NA	NA	NA	Elevated	NA
	1	M	TBD (*TERT*)	NA	35y	NA	NA	NA	NA	NA	Elevated	Shunt resolved after LT
	1	M	TBD (*RTEL1*)	NA	49y	NA	NA	NA	NA	NA	NA	NA
Apostolov R. (20), 2019	1	F	CVID	NA	55y	138	Yes	Yes	NA	NA	NA	Shunt resolved after LT
De La Garza-Ramos C. (21), 2021	1	F	NA	16	53y	NA	NA	NA	NA	NA	NA	Shunt resolved after LT

Johnson G. (22), 2021	1	M	Autoimmune lympho-proliterative syndrome	NA	34y	NA	Yes	Yes	NA	48	NA	Shunt resolved after LT
Hercun J. (23), 2022	2	M	CVID	NA	48y	NA	Yes	NA	NA	NA	NA	LT, recurrence of liver disease 5 months after LT, death
		F	CVID	NA	38y	NA	Yes	NA	NA	NA	NA	LT, recurrence of liver disease 5 years after LT
Khatoon N. (24), 2023	2	NA	NA	NA	NA	NA	NA	NA	NA	NA	NA	LT
Mull E. (25), 2023	1	M	NA	5	13y	NA	NA	Yes	NA	62	48	LT, 0.2L/min oxygen supplementation for activity
Magaz M. (26), 2023	13	NA	NA	NA	NA	NA	NA	NA	NA	NA	NA	LT

PSVD definition dating back to 2019, we included in this table studies meeting the criteria of PSVD but also of idiopathic portal hypertension (27).
NA: data not available.
Abbreviations: CVID, common variable immune deficiency; EV, esophageal varices; LT, liver transplantation; LTOT, long-term oxygen therapy; TBD, telomere biology disorder.

Table S2. List of participating centres and number of patients included.

Hospital, City	Number of patients included
Hôpital Beaujon, Clichy, France	148
Centre Hospitalier Universitaire de Tours, France	13
Centre Hospitalier Universitaire Charles Nicolle, Rouen, France	7
Hôpital Cochin, Paris, France	6
Hôpital Saint-Antoine, Paris, France	5
Centre Hospitalier Universitaire d'Angers, France	3
Hôpital de La Pitié-Salpêtrière, Paris, France	2
Hôpital Saint-Louis, Paris, France	2
Hôpital Bichat Claude-Bernard, Paris, France	2
Hôpital Avicenne, Bobigny, France	1
Centre Hospitalier Universitaire de Grenoble-Alpes, France	1
Centre Hospitalier Universitaire de Rennes, France	1
Hôpital Foch, Suresnes, France	1
Centre Hospitalier Universitaire de Reims, France	1
Centre Hospitalier Universitaire de Brest, France	1
Centre Hospitalier Universitaire de Caen, France	1
Centre Hospitalier Universitaire de Besançon, France	1

Table S3. Characteristics at PSVD diagnosis of the patients with CE-TTE during follow-up, included into the present study, *vs.* those without CE-TTE during follow-up, not included into the present study.

	Patients without CE-TTE (n = 109)		Patients with CE-TTE (n = 196)		*p* value
	n =	Number (%) or median (IQR)	n =	Number (%) or median (IQR)	
Age at diagnosis of PSVD (years)	103	59 (43-67)	187	50 (36-62)	**0.03**
Male sex	109	63 (58)	196	109 (56)	0.71
Laboratory data at diagnosis of PSVD					
Platelet count (x10^9/L)	65	103 (66-150)	155	106 (72-161)	0.94
Prothrombin index (%)	58	86 (73-100)	151	85 (69-97)	0.29
International normalized ratio	46	1.07 (1-1.22)	142	1.10 (1-1.22)	0.65
Serum AST (IU/L)	43	41 (27-63)	149	35 (25-46)	0.13
Serum ALT (IU/L)	43	38 (18-53)	150	32 (20-45)	0.54
Serum ALK (IU/L)	43	129 (97-209)	136	103 (68-146)	**0.018**
Serum GGT (IU/L)	43	89 (48-221)	146	66 (32-132)	**0.021**
Serum total bilirubin (µmol/L)	54	14 (9-24)	149	13 (9-21)	0.80
Serum creatinine (µmol/L)	57	79 (66-116)	147	70 (60-82)	**0.02**
Serum albumin (g/L)	35	37 (31-40)	132	38 (35-42)	0.07
Ascites at diagnosis of PSVD	109	26 (24)	193	9 (5)	**< 0.001**
Hepatic encephalopathy at diagnosis of PSVD	109	1 (1)	193	1 (1)	1
Esophageal or gastric varices at diagnosis of PSVD	95	62 (65)	130	93 (72)	0.32
HVPG at diagnosis of PSVD (mmHg)	82	8 (5-13)	104	6 (3-9)	**0.005**
Liver stiffness at diagnosis of PSVD (kPa)	38	9.15 (6.12-14.8)	108	7.5 (6.1-11.2)	0.18
At least one extrahepatic condition associated with PSVD	109	91 (84)	193	132 (68)	**0.003**

Data are presented as median (interquartile range) or number (proportion) as appropriate. Comparisons of quantitative and qualitative variables were made using Mann-Whitney test and Chi2 or Fisher's exact tests, respectively. Bolded values indicate statistically significant differences ($p \leq 0.05$).
Abbreviations: ALK, alkaline phosphatase; ALT, alanine aminotransferase; AST, aspartate aminotransferase; CE-TTE, contrast-enhanced transthoracic echocardiography; GGT, gamma-glutamyl transpeptidase; HPS, hepatopulmonary syndrome; HVPG, hepatic venous pressure gradient; PSVD, porto-sinusoidal vascular disease.

Table S4. Detailed extrahepatic condition associated with PSVD.

Immunological disorder	HIV infection	Medication or toxin	Hematological disease and prothrombotic condition	Genetic disorder
○ Immune deficiency (n = 38)<br>○ Inflammatory bowel disease (n = 12)<br>○ Sarcoidosis (n = 5)<br>○ Sjögren's syndrome (n = 4)<br>○ Systemic lupus erythematous (n = 3)<br>○ Scleroderma (n = 3)<br>○ History of solid organ transplantation (n = 2)<br>○ Rheumatoid arthritis (n = 1)<br>○ Coeliac disease (n = 1)<br>○ Still disease (n = 1)<br>○ Biermer's disease (n = 0)<br>○ Horton's disease (n = 0)	○ n = 20	○ Azathioprine (n = 13)<br>○ Others (n = 10)<br>○ Chemotherapy including platinum salts (n = 4)<br>○ Methotrexate (n = 2)	○ Anti-phospholipid syndrome (n = 2)<br><br>Thrombophilia (n = 18):<br>○ Factor II or V gene mutations (n = 8)<br>○ Protein C or S deficiency (n = 7)<br>○ Hyperhomocysteinemia (n = 3)<br>○ Antithrombin deficiency (n = 0)<br><br>Myeloproliferative neoplasm (n = 8):<br>○ Polycythema vera (n = 6)<br>○ Myelofibrosis (n = 2)<br><br>○ Myelodysplastic syndrome (n = 3)<br><br>Lymphoproliferative syndrome (n = 19):<br>○ B-cell lymphoma (n = 7)<br>○ Monoclonal gammapathy of undetermined significance (n = 4)<br>○ Hodgkin lymphoma (n = 2)<br>○ Acute lymphocytic leukemia (n = 2)<br>○ Castleman disease or TAFRO syndrome (n = 2)<br>○ Myeloma (n = 1)<br>○ Chronic lymphocytic leukemia (n = 1)	○ Telomere biology disorder (n = 13)<br>○ Pierpont syndrome (n = 1)<br>○ Noonan syndrome (n = 1)<br>○ Coats-plus syndrome (n = 1)<br>○ Unknown genetic disease (n = 1)

132 patients had an extrahepatic condition associated with PSVD: 1 in 90 patients, 2 in 34 patients, 3 in 6 patients, 4 in 1 patient, and 5 in 1 patient.
Abbreviations: HIV, human immunodeficiency virus infection; INR, international normalized ratio; PSVD, porto-sinusoidal vascular disorder.

Table S5. Characteristics at the time of CE-TTE of the patients with HPS with pulmonary fibrosis, *vs.* those without pulmonary fibrosis.

	Patients with HPS and without pulmonary fibrosis (n =11)		Patients with HPS and pulmonary fibrosis (n = 3)		*p* value
	n =	Number (%) or median (IQR)	n =	Number (%) or median (IQR)	
Age at diagnosis of PSVD (years)	10	37 (31-59)	3	54 (44-55)	0.74
Age at the time of CE-TTE (years)	11	42 (36-57)	3	56 (45-60)	0.81
Duration between diagnosis of PSVD and first CE-TTE (months)	10	36 (1-100)	3	6 (3-68)	0.71
Male sex	11	7 (64)	3	3 (100)	0.50
Body mass index (kg/m^2)	11	23 (21-28)	3	23 (22-24)	
Current or past smoking	11	5 (45)	3	2 (67)	0.44
History of excessive alcohol consumption	11	1 (1)	3	0 (0)	>0.9
Metabolic comorbidities[a]	11	5 (45)	3	1 (33)	>0.9
At least one extrahepatic condition associated with PSVD	11	8 (73)	3	2 (67)	>0.9
Laboratory data					
Hemoglobin (g/dL)	9	11.7 (9.9-12.3)	3	14.7 (14.3-15.2)	**0.04**
Platelet count (x10^9/L)	10	106 (79-125)	3	101 (74-119)	>0.9
Prothrombin index (%)	10	62 (61-79)	3	86 (72-89)	0.55
INR	10	1.3 (1.1-1.4)	3	1.1 (1.1-1.2)	0.55
Serum AST (IU/L)	10	43 (30-66)	3	44 (43-77)	0.57
Serum ALT (IU/L)	10	22 (17-23)	3	42 (32-61)	0.075
Serum ALK (IU/L)	10	184 (163-257)	3	138 (135-222)	0.69
Serum GGT (IU/L)	10	87 (41-144)	3	51 (46-61)	0.93
Serum total bilirubin (µmol/L)	10	46 (32-72)	3	24 (16-28)	0.11
Serum creatinine (µmol/L)	10	58 (43-63)	3	81 (67-83)	0.47
Serum albumin (g/L)	8	32 (28-34)	2	29 (28-32)	0.59
Signs of portal hypertension					
Thrombocytopenia	10	9 (90)	3	3 (100)	>0.9
Ascites	11	1 (9)	3	0 (0)	>0.9
Splenomegaly	11	10 (91)	3	2 (67)	0.06
Portosystemic collaterals at imaging	11	10 (91)	3	3 (100)	>0.9
Small esophageal varices	10	2 (20)	3	0 (0)	>0.9
Gastric or large esophageal varices, or history of variceal band ligation	11	7 (64)	3	2 (67)	>0.9
Vibration controlled transient elastography					
Liver stiffness (kPa)	5	21.5 (11.7-24.6)	2	5.6 (5.3-5.8)	0.09
Spleen stiffness (kPa)	4	46.6 (35-55)	1	54.1 (54.1-54.1)	>0.9
Complications of PSVD until CE-TTE					
History of ascites	11	2 (18)	3	0 (0)	>0.9
History of hepatic encephalopathy	11	1 (9)	3	0 (0)	>0.9
History of esophageal or gastric variceal bleeding	11	0 (0)	3	0 (0)	1
History of portal vein and/or left or right branches thrombosis	11	2 (18)	3	0 (0)	>0.9
History of mesenteric venous and/or splenic vein thrombosis	11	1 (9)	3	0 (0)	>0.9
Medications					
Anticoagulation therapy	11	1 (9)	3	0 (0)	>0.9
Diuretic therapy	11	3 (27)	3	0 (0)	>0.9
Respiratory features					
$AaPO_2$ (mmHg) at the time of CE-TTE	10	31 (27-40)	3	18 (17-36)	0.49
LTOT	11	5 (45)	3	2 (67)	>0.9
Liver transplantation	11	4 (36)	3	1 (33)	>0.9
Death	11	3 (27)	3	1 (33)	>0.9

Data are presented as median (interquartile range) or number (proportion) as appropriate. Comparisons of quantitative and qualitative variables were made using
Mann-Whitney test and Chi2 or Fisher's exact tests, respectively. Bolded values indicate statistically significant differences ($p < 0.05$).
[a] Metabolic comorbidities included overweight (BMI ≥ 25 kg/m^2), diabetes mellitus, arterial hypertension, and/or dyslipidemia.
Abbreviations: $AaPO_2$, alveolar-arterial oxygen gradient; ALK, alkaline phosphatase; ALT, alanine aminotransferase; AST, aspartate aminotransferase; CE-TTE, contrast-enhanced transthoracic echocardiography; GGT, gamma-glutamyl transpeptidase; HPS, hepatopulmonary syndrome; INR, international normalized ratio; LTOT, long-term oxygen therapy; PSVD, porto-sinusoidal vascular disorder.

Table S6. Histological features at the time of diagnosis of PSVD after reviewing by an expert pathologist.

	PSVD without HPS (n = 103) Number (%) or median (IQR)	PSVD with HPS (n = 9) Number (%) or median (IQR)	*p*-value
Biopsy length (mm)	25 (20-34)	25 (20-38)	0.8
Number of portal tract	10 (7-14)	8 (7-12)	0.6
Obliterative portal venopathy *(obliteration of portal vein branches; thickening of the wall)*			0.085
Absence	54 (55)	2 (22)	
Incomplete or complete	45 (45)	7 (78)	
Herniated portal vein *(a portal vein from the portal tract directly abutting periportal parenchyma)*	35 (35)	2 (39)	0.9
Hypervascularised portal tract *(multiple thin-walled vascular spaces in the portal tract)*	19 (19)	1 (14)	0.9
Periportal abnormal vessels *(single or multiple thin-walled vascular spaces of different caliber outside, but in close contact with, the portal tract)*	15 (15)	1 (14)	0.9
Nodular regenerative hyperplasia			0.069
Normal architecture	66 (67)	3 (33)	
Slight nodulation or nodular regenerative hyperplasia	33 (33)	6 (67)	
Incomplete septal cirrhosis	0 (0)	0 (0)	-
Sinusoidal dilatation			0.9
Absence	36 (36)	3 (33)	
Presence (mild or severe)	64 (64)	6 (67)	
Peri sinusoidal fibrosis (mild or severe)	5 (5)	1 (11)	0.42
Sinusoidal congestion	7 (7)	1 (14)	0.4
Steatosis			0.2
S0 (< 5%)	78 (80)	9 (100)	
Present (5% or more)	20 (19)	0 (0)	
Fibrosis stage ≥ F1	43 (43)	6 (67)	0.2

Data are presented as median (interquartile range) or number (proportion) as appropriate. Comparisons of quantitative and qualitative variables were made using Mann-Whitney test and Chi2 or Fisher's exact tests, respectively. Bolded values indicate statistically significant differences ($p < 0.05$).
Abbreviations: HPS, hepatopulmonary syndrome; PSVD, porto sinusoidal vascular disorder.

Table S7. Characteristics at the time of CE-TTE of the patients with HPS with symptoms, *vs.* those without symptoms.

	Patients with HPS without respiratory symptom (n = 4)		Patients with HPS with respiratory symptoms (n = 10)		*p* value
	n =	Number (%) or median (IQR)	n =	Number (%) or median (IQR)	
Age at diagnosis of PSVD (years)	4	45 (32-58)	9	38 (33-54)	0.71
Age at the time of CE-TTE (years)	4	49 (41-58)	10	42 (35-61)	0.6
Duration between diagnosis of PSVD and first CE-TTE (months)	4	48 (4-103)	9	31 (1-107)	>0.9
Male sex	4	3 (75)	10	7 (70)	1
Body mass index (kg/m^2)	4	25.9 (24.1-27.7)	10	22.4 (20.5-24.5)	0.3
Current or past smoking	4	1 (25)	10	6 (60)	0.6
History of excessive alcohol consumption	4	1 (25)	10	0 (0)	0.3
Metabolic comorbidities[a]	4	3 (75)	10	3 (30)	0.2
At least one extrahepatic condition associated with PSVD [b]	4	1 (25)	10	9 (90)	**0.041**
Laboratory data					
Hemoglobin (g/dL)	4	12.8 (10.1-15.6)	9	12.1 (11.3-13.6)	0.61
Platelet count (x10^9/L)	4	109 (83-120)	9	98 (74-128)	>0.9
Prothrombin index (%)	4	61 (58-69)	9	75 (61-84)	0.3
INR	4	1.3 (1.2-1.4)	9	1.2 (1.1-1.3)	0.35
Serum AST (IU/L)	4	55 (38-78)	9	44 (37-59)	>0.9
Serum ALT (IU/L)	4	33 (23-47)	9	22 (16-23)	0.3
Serum ALK (IU/L)	4	160 (115-239)	9	181 (160-266)	0.7
Serum GGT (IU/L)	4	62 (46-104)	9	100 (40-155)	>0.9
Serum total bilirubin (µmol/L)	4	46 (35-49)	9	32 (24-79)	>0.9
Serum creatinine (µmol/L)	4	74 (62-95)	9	52 (40-61)	0.076
Serum albumin (g/L)	3	33 (25-35)	7	32 (29-33)	0.73
Signs of portal hypertension					
Thrombocytopenia	4	4 (100)	10	9 (90)	>0.9
Ascites	4	0 (0)	10	1 (1)	>0.9
Splenomegaly	4	4 (100)	10	8 (80)	>0.9
Portosystemic collaterals at imaging	4	4 (100)	10	9 (90)	>0.9
Small esophageal varices	4	1 (25)	9	1 (11)	>0.9
Gastric or large esophageal varices, or history of variceal band ligation	4	3 (75)	10	6 (60)	>0.9
Vibration controlled transient elastography					
Liver stiffness (kPa)	3	8.3 (7.2-14.9)	4	18.2 (10.0-33.7)	0.63
Spleen stiffness (kPa)	1	54.1 (54.1-54.1)	4	46.6 (35.1-54.9)	1
Complications of PSVD until CE-TTE					
History of ascites	4	1 (25)	10	1 (10)	0.5
History of hepatic encephalopathy	4	1 (25)	10	1 (25)	0.5
History of esophageal or gastric variceal bleeding	4	0 (0)	10	1 (10)	>0.9
History of portal vein and/or left or right branches thrombosis	4	2 (50)	10	0 (0)	0.066
History of mesenteric venous and/or splenic vein thrombosis	4	1 (25)	10	0 (0)	0.3
Medications					
Anticoagulation therapy	4	1 (25)	10	0 (0)	0.3
Diuretic therapy	4	2 (50)	10	1 (10)	0.2
Respiratory features					
$AaPO_2$ (mmHg) at the time of CE-TTE	4	20 (15-25)	9	35 (30-50)	**0.017**
LTOT	4	1 (25)	10	6 (60)	0.6
Liver transplantation	4	1 (25)	10	4 (40)	>0.9
Death	4	1 (25)	10	3 (30)	>0.9

Data are presented as median (interquartile range) or number (proportion) as appropriate. Comparisons of quantitative and qualitative variables were made using
Mann-Whitney test and Chi2 or Fisher's exact tests, respectively. Bolded values indicate statistically significant differences ($p < 0.05$).
[a] Metabolic comorbidities included overweight (BMI ≥ 25 kg/m^2), diabetes mellitus, arterial hypertension, and/or dyslipidemia.
[b] One patient without symptoms had at least one extrahepatic condition associated with PVSD (Patient 10), versus 9 patients with symptoms (Patients 2, 3, 5, 6, 7, 9, 12, 13, and 14).
Abbreviations: $AaPO_2$, alveolar-arterial oxygen gradient; ALK, alkaline phosphatase; ALT, alanine aminotransferase; AST, aspartate aminotransferase; CE-TTE, contrast-enhanced transthoracic echocardiography; GGT, gamma-glutamyl transpeptidase; HPS, hepatopulmonary syndrome; INR, international normalized ratio; LTOT, long-term oxygen therapy; PSVD, porto-sinusoidal vascular disorder.

Table S8. Univariate analysis of variables at CE-TTE predicting cumulative incidence of LT not related to HPS or of death, in patients with PSVD (Fine and Gray model).

	Univariate analysis			
	n =	HR	95% CI	*p* value
Hepatopulmonary syndrome	196	1.84	0.58, 5.80	0.3
Male sex	196	2.56	0.90, 7.29	0.078
Age	196	1.02	0.99, 1.06	0.3
BMI > 25 kg/m^2	195	1.62	0.64, 4.10	0.3
Tobacco exposure	194	0.96	0.34, 2.68	>0.9
Laboratory data				
Hemoglobin (g/dL)	176	0.75	0.56, 1.01	0.058
Platelets count (G/L)	183	1.00	0.99, 1.01	>0.9
Prothrombin index (%)	175	0.99	0.97, 1.01	0.2
INR	177	1.62	0.55, 4.78	0.4
AST (IU/L)	182	1.01	1.01, 1.02	**<0.001**
ALT (IU/L)	185	1.00	0.98, 1.02	0.8
ALK (IU/L)	154	1.00	1.00, 1.00	0.5
GGT (IU/L)	184	1.00	0.99, 1.00	0.3
Serum total bilirubin (µmol/L)	185	1.02	1.00, 1.03	**0.024**
Serum creatinine (µmol/L)	170	1.01	0.99, 1.04	0.2
Serum albumin (g/L)	170	0.93	0.86, 1.01	0.10
Signs of portal hypertension				
Thrombocytopenia	195	1.07	0.38, 3.01	0.9
Ascites until/at CE-TTE	195	7.00	2.78, 17.6	**<0.001**
Splenomegaly	191	2.16	0.49, 9.55	0.3
Portosystemic collaterals at imaging	192	1.71	0.49, 5.95	0.4
Presence of varices	186	2.54	0.82, 7.87	0.11
Varices at risk	191	5.91	1.36, 25.7	**0.018**
Hepatic encephalopathy	196	1.25	0.14, 11.3	0.8
Liver stiffness (kPa)	167	1.04	1.00, 1.08	**0.045**
Spleen stiffness (kPa)	95	1.02	0.99, 1.05	0.2
Portal venous thrombosis	196	0.97	0.28, 3.36	>0.9
Mesenteric or splenic venous thrombosis	196	0.00	0.00, 0.00	**<0.001**

Data are presented as HR and 95% CI. Bolded values indicate statistically significant differences ($p < 0.05$) (Fine and Gray models).

Abbreviations: ALK, alkaline phosphatase; ALT, alanine aminotransferase; AST, aspartate aminotransferase; BMI, body mass index; CI, confidence interval; CE-TTE, contrast-enhanced transthoracic echocardiography; GGT, gamma-glutamyl transpeptidase; HPS, hepatopulmonary syndrome; HR, Hazard ratio; INR, international normalized ratio; LT, liver transplantation; PSVD, porto-sinusoidal vascular disorder.

Table S9. Univariate analysis of variables at CE-TTE predicting cumulative incidence of liver related events or of LT not related to HPS or of liver-related death, in patients with PSVD (Fine and Gray model).

	Univariate analysis			
	n =	HR	95% CI	*p* value
Hepatopulmonary syndrome	196	1.84	0.76, 4.43	0.2
Male sex	196	2.46	1.18, 5.14	**0.016**
Age	196	1.03	1.00, 1.06	**0.025**
BMI > 25 kg/m^2	195	1.62	0.83, 3.17	0.2
Tobacco exposure	194	0.64	0.28, 1.46	0.3
Laboratory data				
Hemoglobin (g/dL)	176	0.84	0.71, 1.00	0.055
Platelets count (G/L)	183	1.00	0.99, 1.00	0.3
Prothrombin index (%)	175	0.98	0.96, 0.99	**0.001**
INR	177	3.97	1.73, 9.11	**0.001**
AST (IU/L)	182	1.01	1.00, 1.01	**0.003**
ALT (IU/L)	185	1.00	0.99, 1.02	0.9
ALK (IU/L)	154	1.00	1.00, 1.00	0.3
GGT (IU/L)	184	1.00	1.00, 1.00	> 0.9
Serum total bilirubin (µmol/L)	185	1.01	1.00, 1.02	**0.015**
Serum creatinine (µmol/L)	170	1.01	0.99, 1.02	0.3
Serum albumin (g/L)	170	0.94	0.89, 0.99	**0.018**
Signs of portal hypertension				
Thrombocytopenia	195	1.33	0.62, 2.85	0.5
Ascites until/at CE-TTE	195	3.98	1.90, 8.35	**< 0.001**
Splenomegaly	191	3.53	1.06, 11.8	**0.041**
Portosystemic collaterals at imaging	192	2.25	0.86, 5.89	0.10
Presence of varices	186	1.48	0.75, 2.95	0.3
Varices at risk	191	9.06	2.83, 29.0	**< 0.001**
Hepatic encephalopathy	196	3.41	1.07, 10.9	**0.038**
Liver stiffness (kPa)	167	1.04	1.00, 1.07	**0.032**
Spleen stiffness (kPa)	95	1.04	1.02, 1.06	**< 0.001**
Portal venous thrombosis	196	2.90	1.45, 5.79	**0.003**
Mesenteric and/or splenic venous thrombosis	196	7.67	2.63, 22.3	**< 0.001**

Data are presented as HR and 95% CI. Bolded values indicate statistically significant differences ($p < 0.05$) (Fine and Gray model).

Abbreviations: ALK, alkaline phosphatase; ALT, alanine aminotransferase; AST, aspartate aminotransferase; BMI, body mass index; CI, confidence interval; CE-TTE, contrast-enhanced transthoracic echocardiography; GGT, gamma-glutamyl transpeptidase; HPS, hepatopulmonary syndrome; INR, international normalized ratio; LT, liver transplantation; PSVD, porto-sinusoidal vascular disorder.

Table S10. Characteristics at the time of plasma collection of patients with cirrhosis with or without HPS randomly selected from the MICROSPY cohort.

	Cirrhosis without HPS (n = 22)		Cirrhosis with HPS (n = 11)		*p* value
	n =	Number (%) or median (IQR)	n =	Number (%) or median (IQR)	
Age (years)	22	56 (52-63)	11	55 (52-59)	0.8
Male sex	22	12 (55)	11	10 (91)	0.054
Cause of cirrhosis					
Alcohol	22	12 (55)	11	6 (55)	> 0.9
Nonalcoholic steatohepatitis	22	6 (27)	11	2 (18)	0.7
Hepatitis C	22	8 (36)	11	2 (18)	0.4
Hepatitis B	22	3 (14)	11	1 (9)	> 0.9
Other	22	1 (5)	11	2 (18)	0.2
Liver test					
Prothrombin index (%)	22	61 (53-73)	11	56 (49-67)	0.4
International normalized ratio	22	1.28 (1.17-1.40)	11	1.38 (1.24-1.50)	0.2
Serum total bilirubin (µmol/L)	22	20 (12-41)	11	36 (31-44)	**0.045**
Serum creatinine (µmol/L)	22	68 (59-76)	11	71 (68-89)	0.3
Serum albumin (g/L)	22	34 (27-37)	11	31 (29-34)	0.7
Ascites	22	8 (36)	11	6 (55)	0.5
Hepatic encephalopathy	22	2 (9.1)	11	5 (45)	**0.027**
Child-Pugh score	22		11		0.2
Child-Pugh A		9 (41)		1 (9.1)	
Child-Pugh B		9 (41)		7 (64)	
Child-Pugh C		4 (18)		3 (27)	
MELD score	22	13 (9-17)	11	14 (13-16)	0.6
HVPG (mmHg)	20	17 (11-20)	10	19 (13-23)	0.4

Data are presented as median (interquartile range) or number (proportion) as appropriate. Comparisons of quantitative and qualitative variables were made using Mann-Whitney test and Chi2 or Fisher's exact tests, respectively. Bolded values indicate statistically significant differences ($p < 0.05$).
Abbreviations: HPS, hepatopulmonary syndrome; HVPG, hepatic venous pressure gradient.

Supplementary references

1. Marchand EJ, De Jesús M, Biascoechea ZAR. Cyanotic syndrome of portal hypertension in hepatosplenic schistosomiasis and portal cirrhosis. The American Journal of Cardiology. oct 1962;10(4):496-506.
2. Babbs C, Warnes TW, Haboubi NY. Non-cirrhotic portal hypertension with hypoxaemia. Gut. 1 janv 1988;29(1):129-31.
3. Krowka MJ, Wiseman GA, Burnett OL, et al. Hepatopulmonary Syndrome. Chest. sept 2000;118(3):615-24.
4. De BK, Sen S, Sanyal R. Hepatopulmonary Syndrome in Noncirrhotic Portal Hypertension. Ann Intern Med. 6 juin 2000;132(11):924.
5. Kaymakoglu S, Kahraman T, Kudat H, et al. Hepatopulmonary syndrome in noncirrhotic portal hypertensive patients. Dig Dis Sci. mars 2003;48(3):556-60.
6. Taille C, Cadranel J, Bellocq A, et al. Liver transplantation for hepatopulmonary syndrome: A ten-year experience in Paris, France. Transplantation. 15 mai 2003;75(9):1482-9.
7. Swanson KL, Wiesner RH, Krowka MJ. Natural history of hepatopulmonary syndrome: Impact of liver transplantation. Hepatology. mai 2005;41(5):1122-9.
8. Krasinskas AM, Eghtesad B, Kamath PS, et al. Liver transplantation for severe intrahepatic noncirrhotic portal hypertension. Liver Transpl. juin 2005;11(6):627-34.
9. Yilmaz S, Dursum M, Canoruç F, et al. A severe (type II) hepatopulmonary syndrome in a patient with idiopathic portal hypertension and treatment with paroxetine. Neth J Med. déc 2005;63(11):448-52.
10. Deibert P, Allgaier HP, Loesch S, et al. Hepatopulmonary syndrome in patients with chronic liver disease: role of pulse oximetry. BMC Gastroenterol. déc 2006;6(1):15.
11. Gupta S, Castel H, Rao RV, et al. Improved Survival After Liver Transplantation in Patients with Hepatopulmonary Syndrome. American Journal of Transplantation. févr 2010;10(2):354-63.
12. Maganty K, Ghanta R, Bejarano P, et al. Liver Transplantation for Hepatopulmonary Syndrome Due to Noncirrhotic Portal Hypertension. Transplantation Proceedings. sept 2011;43(7):2814-6.
13. Cazals-Hatem D, Hillaire S, Rudler M, et al. Obliterative portal venopathy: Portal hypertension is not always present at diagnosis. Journal of Hepatology. mars 2011;54(3):455-61.
14. Naalsund A, Lund MB, Mynarek G, et al. En mann i 60-årene med alvorlig respirasjonssvikt. Tidsskriftet. 2011;131(17):1654-7.
15. Cantez MS, Gerenli N, Ertekin V, et al. Hepatoportal Sclerosis in Childhood: Descriptive Analysis of 12 Patients. J Korean Med Sci. 2013;28(10):1507.
16. Franchi-Abella S, Fabre M, Mselati E, et al. Obliterative Portal Venopathy: A Study of 48 Children. The Journal of Pediatrics. juill 2014;165(1):190-193.e2.
17. Alhosh R, Genyk Y, Alexopoulos S, et al. Hepatopulmonary syndrome associated with nodular regenerative hyperplasia after liver transplantation in a child. Pediatr Transplantation. août 2014;18(5):E157-60.
18. Holmes SN, Condliffe A, Griffiths W, et al. Familial Hepatopulmonary Syndrome in Common Variable Immunodeficiency. J Clin Immunol. avr 2015;35(3):302-4.
19. Gorgy AI, Jonassaint NL, Stanley SE, et al. Hepatopulmonary Syndrome Is a Frequent Cause of Dyspnea in the Short Telomere Disorders. Chest. oct 2015;148(4):1019-26.
20. Apostolov R, Sinclair M, Lokan J, et al. Successful liver transplantation in common variable immune deficiency with reversal of hepatopulmonary syndrome. BMJ Case Rep. avr 2019;12(4):e226095.

21. De La Garza-Ramos C, Muneer MS, Lewis JT, et al. Transportal Technetium-99m Labeled Macroaggregated Albumin Scintigraphy to Quantify Occult Intrahepatic Microvascular Portosystemic Shunting. Radiology Case Reports. avr 2021;16(4):975-8.

22. Johnson G, Huber A, Levstik M, et al. S2660 A Case of Hepatopulmonary Syndrome Requiring Living Donor Liver Transplantation in a Patient With Nodular Regenerative Hyperplasia and Autoimmune Lymphoproliferative Syndrome-Associated Granulomatous Hepatitis Without Cirrhosis. Am J Gastroenterol. oct 2021;116(1):S1115-S1115.

23. Hercun J, Parikh E, Kleiner DE, et al. Recurrent Nodular Regenerative Hyperplasia Following Liver Transplantation in Common Variable Immunodeficiency. Hepatology. sept 2021;74(3):1698-701.

24. Khatoon N, Keaveny AP, Carames GP, et al. Hepatoportal Sclerosis—A Clinicopathologic Review of 28 Cases. Gastro Hep Advances. 2023;2(3):303-6.

25. Mull E, Ronau R, Adler B, et al. Hepatopulmonary Syndrome with Noncirrhotic Portal Hypertension Diagnosed Following Acute SAR-CoV-2 Infection [Internet]. Preprints; 2023 mai [cité 11 juin 2023]. Disponible sur: https://www.authorea.com/users/326440/articles/642592-hepatopulmonary-syndrome-with-noncirrhotic-portal-hypertension-diagnosed-following-acute-sar-cov-2-infection?commit=5d805fa50af09204d2fa276c2cdcac3c1336460a

26. Magaz M, Giudicelli-Lett H, Rajoriya N, et al. Liver Transplant for Porto-sinusoidal Vascular Disease: Long-term Outcome. HPB. 2021;23:S950-1.

27. De Gottardi A, Rautou PE, Schouten J, et al. Porto-sinusoidal vascular disease: proposal and description of a novel entity. The Lancet Gastroenterology & Hepatology. mai 2019;4(5):399-411.
