# Supplementary material for: Hepatopulmonary syndrome in patients with porto-sinusoidal vascular disorder: Characteristics and outcome
Source: JHEP Rep. 2024 Dec 20;7(4):101310. doi: 10.1016/j.jhepr.2024.101310 (PMC11960633; doi:10.1016/j.jhepr.2024.101310)
Supplement: Multimedia component 2 [file mmc2.docx]

**JHEP Reports**

**CTAT methods**

**Tables for a “Complete, Transparent, Accurate and Timely account” (CTAT) are now mandatory for all revised submissions. The aim is to enhance the reproducibility of methods.**

- **Only include the parts relevant to your study**
- **Refer to the CTAT in the main text as ‘Supplementary CTAT Table’**
- **Do not add subheadings**
- **Add as many rows as needed to include all information**
- **Only include one item per row**

**If the CTAT form is not relevant to your study, please outline the reasons why:**

|  |
| --- |

- 1. **Antibodies**

| **Name** | **Citation** | **Supplier** | **Cat no.** | **Clone no.** |
| --- | --- | --- | --- | --- |
| **Not applicable** | | | | |

- 1. **Cell lines**

| **Name** | **Citation** | **Supplier** | **Cat no.** | **Passage no.** | **Authentication test method** |
| --- | --- | --- | --- | --- | --- |
| **Not applicable** | | | | | |

- 1. **Organisms**

| **Name** | **Citation** | **Supplier** | **Strain** | **Sex** | **Age** | **Overall n number** |
| --- | --- | --- | --- | --- | --- | --- |
| **Not applicable** | | | | | | |

- 1. **Sequence based reagents**

| **Name** | **Sequence** | **Supplier** |
| --- | --- | --- |
| **Not applicable** | | |

- 1. **Biological samples**

| **Description** | **Source** | **Identifier** |
| --- | --- | --- |
| For all patients, peripheral venous blood was collected from the cubital vein, with a tourniquet needle, in 0.129 mol/L citrated tubes. Two successive centrifugations were performed, each of 15 min at 2500 g at 20°C. Aliquots of platelet-free plasma were then stored at −80 °C until use.  previously published MICROSPY cohort.  According to the manufacturer’s instructions we measured concentrations (pg/mL) of angiopoietin 2 (DY623 DY008; R&D Systems Europe, France), Tie2 (DY5159, DY008; R&D Systems Europe, France), ICAM3 (DY715, DY008, R&D Systems Europe, France), VCAM1 (DY809-05, DY008; R&D Systems Europe, France), IL-6 (DY206-05, DY008; R&D Systems Europe, France), and TNF-α (DY210-05, DY008; R&D Systems Europe, France). The chromogenic limulus amoebocyte lysate assay (Endochrome-K test R1708K; Charles River Laboratories, Charleston, South Carolina) was used for the detection of endotoxin. For optimal test results, platelet-free plasmas were diluted 1:10 with endotoxin-free water and heat treated for 30 min at 75°C. Samples were then mixed with limulus amoebocyte lysate reagent and absorbance of the plate (405 nm) then read for 1 hour using a kinetic microplate reader (Tecan Spark 10M) and analyzed. To reduce inter-assay variability, all samples were measured as a single batch. | For patient with PSVD, blood was collected at the time of CE-TTE or within 12 months before or after CE-TTE. Patients with cirrhosis were identified within the previously published MICROSPY cohort (DOI: [10.1002/hep.29903](https://doi.org/10.1002/hep.29903)). | Each patient was given a unique identifying study code and on storage of blood samples these were barcoded with this unique identifier code to anonymise the sample to any persons conducting downstream sample processing. |

- 1. **Deposited data**

| **Name of repository** | **Identifier** | **Link** |
| --- | --- | --- |
|  | | |

- 1. **Software**

| **Software name** | **Manufacturer** | **Version** |
| --- | --- | --- |
| **SPSS** | **SPSS Inc, Chicago, IL** | **29.0** |
| **R studio** | **www.r-project.org** | **4.0.2.** |
| **Excel** | **Microsoft, Redmond, Washington** | **16.37** |

- 1. **Other (*e.g*. drugs, proteins, vectors etc.)**

| **Not applicable** | | |
| --- | --- | --- |
|  |  |  |

- 1. **Please provide the details of the corresponding methods author for the manuscript:**

| **Dre. Sabrina SIDALI, MD, PhD student**  Service d’Hépatologie, Hôpital Beaujon, Assistance Publique des Hôpitaux de Paris,  100 Boulevard du Général Leclerc, 92110 Clichy, France  Telephone: +33 1 40 87 55 01  Fax: +33 1 40 87 55 30  E-mail: [sidali.sabrina@gmail.com](mailto:sidali.sabrina@gmail.com)  **Prof. Pierre-Emmanuel RAUTOU, MD, PhD**  Service d’Hépatologie, Hôpital Beaujon, Assistance Publique des Hôpitaux de Paris,  100 Boulevard du Général Leclerc, 92110 Clichy, France  Telephone: +33 1 40 87 55 01  Fax: +33 1 40 87 55 30  E-mail: [pierre-emmanuel.rautou@inserm.fr](mailto:pierre-emmanuel.rautou@inserm.fr) |
| --- |

**2.0 Please confirm for randomised controlled trials all versions of the clinical protocol are included in the submission. These will be published online as supplementary information.**

| **Not applicable** |
| --- |
